# Cell-based chemical fingerprinting identifies telomeres and lamin A as modifiers of DNA damage response in cancer cells

**DOI:** 10.1038/s41598-018-33139-x

**Published:** 2018-10-04

**Authors:** Chiaki Fujiwara, Yukiko Muramatsu, Megumi Nishii, Kazuhiro Tokunaka, Hidetoshi Tahara, Masaru Ueno, Takao Yamori, Yoshikazu Sugimoto, Hiroyuki Seimiya

**Affiliations:** 1 Division of Molecular Biotherapy, Cancer Chemotherapy Center, Japanese Foundation for Cancer Research, Tokyo, 135–8550 Japan; 2 Division of Chemotherapy, Faculty of Pharmacy, Keio University, Tokyo, 105–8512 Japan; 3 Department of Molecular Biotechnology, Graduate School of Advanced Sciences of Matter, Hiroshima University, Higashi-Hiroshima, 739–8530 Japan; 4 Biomedicine Group, Pharmaceutical Research Laboratories, Pharmaceuticals Group, Nippon Kayaku Co., Ltd., Tokyo, 115–8588 Japan; 5 Department of Cellular and Molecular Biology, Division of Integrated Medical Science, Graduate School of Biomedical Sciences, Hiroshima University, Hiroshima, 734–8551 Japan; 6 Division of Molecular Pharmacology, Cancer Chemotherapy Center, Japanese Foundation for Cancer Research, Tokyo, 135–8550 Japan; 7 Present Address: Pharmaceuticals and Medical Devices Agency, Tokyo, 100-0013 Japan

## Abstract

Telomere maintenance by telomerase activity supports the infinite growth of cancer cells. MST-312, a synthetic telomerase inhibitor, gradually shortens telomeres at non-acute lethal doses and eventually induces senescence and apoptosis of telomerase-positive cancer cells. Here we report that MST-312 at higher doses works as a dual inhibitor of telomerase and DNA topoisomerase II and exhibits acute anti-proliferative effects on cancer cells and xenografted tumours *in vivo*. Our cell-based chemical fingerprinting approach revealed that cancer cells with shorter telomeres and lower expression of lamin A, a nuclear architectural protein, exhibited higher sensitivity to the acute deleterious effects of MST-312, accompanied by formation of telomere dysfunction-induced foci and DNA double-strand breaks. Telomere elongation and lamin A overexpression attenuated telomeric and non-telomeric DNA damage, respectively, and both conferred resistance to apoptosis induced by MST-312 and other DNA damaging anticancer agents. These observations suggest that sufficient pools of telomeres and a nuclear lamina component contribute to the cellular robustness against DNA damage induced by therapeutic treatment in human cancer cells.

## Introduction

Telomeres are protective structures at the ends of eukaryotic linear chromosomes that consist of telomeric DNA, (TTAGGG)n, and binding proteins, such as shelterin^1^. Telomeric DNA has a single-stranded 3′-overhang that forms a protective t-loop structure^2^. Because the DNA polymerase-dependent replication machinery cannot replicate the ends of linear DNAs (the end replication problem), telomeres shorten after each round of DNA replication. Critically shortened telomeres lose the ability to form the t-loop structure and are then recognised as deleterious DNA double-strand breaks^3^. This condition leads to a block in DNA replication and cell division, resulting in a permanent growth arrest, called replicative senescence. Thus, in normal cells, telomere shortening and the accompanying inhibition of cell division blocks the growth of cells and functions as a tumour suppressor mechanism^4^. In contrast, dysfunction of telomeres can drive genomic instability, which can facilitate tumour progression^5^.

In vast majority of human cancer cells, the end replication problem is solved by the maintenance of telomeres by the telomerase enzyme^6^, which consists of the hTERT catalytic subunit and the RNA template (called hTR or hTERC, in humans). While hTR is ubiquitously expressed in broad ranges of tissues, hTERT is the limiting factor for telomerase activity^7^. Recent cancer genome analyses have identified hTERT promoter mutations that lead to elevated transcriptional activation of the gene in many cancers^8,9^. These cancer cells harbouring mutations in hTERT acquire unlimited proliferative potential^10^. Therefore, telomerase has been postulated as a therapeutic target for cancer. Indeed, various telomerase inhibitors, including epigallocatechin gallate (EGCG), a major tea catechin, and its synthetic derivative, MST-312, have been found or developed^11–15^. These inhibitors gradually shorten telomeres in cancer cells to eventually induce cell crisis^11–16^. Among them, imetelstat is currently under clinical investigation^17–19^.

In general, most anticancer drugs exhibit rapid anti-proliferative activities in cancer cells. However, telomerase inhibitors are an exception, for the following reasons. First, because the end replication problem only occurs during DNA synthesis, telomere shortening depends on cell growth. Therefore, the process of inhibiting telomerase to allow for telomere shortening requires the treatment of cancer cells with telomerase inhibitors at low concentrations that do not induce inhibition of cell growth. In the presence of telomerase inhibitors at such low concentrations, cancer cells would continue to grow but their telomeres would gradually shorten, suggesting that the anti-proliferative effect of telomerase inhibitors on cancer cells would only be seen after the shortened telomeres lose their end-capping functions. This suggests that the initial telomere length and the rate of telomere shortening would determine the duration of the inhibitor treatment required for the induction of telomere crisis in target cells. We previously showed that elevated expression of a telomeric poly(ADP-ribose) polymerase (PARP) tankyrase 1, which enhances telomerase access to telomeres^20,21^, blocks efficient telomere shortening by the telomerase inhibitor MST-312 at non-toxic low doses and confers resistance to the compound^16^. Conversely, tankyrase 1 blockade by PARP inhibitors enhances telomere shortening by MST-312 and induces earlier crisis of cells^16^.

Interestingly, RNA interference-mediated knockdown of the hTR template RNA component of telomerase results in a rapid deleterious effect on cancer cell growth^22,23^. This effect is observed independently of telomere length and is not accompanied by telomere shortening, suggesting a telomere-independent mechanism in blocking cell growth. Furthermore, accumulating evidence has shown that telomerase supports tumourigenesis and normal stem cell growth in a telomere-independent manner^24–26^. These observations suggest that human cancer cells are “addicted” to telomerase activity even if they have sufficiently long telomeres. By contrast, telomerase knockout mice can be born and grow fertile until the telomeres shorten over generations to reach the minimal length that is required for chromosome end capping^27^. Together this suggests that a telomerase inhibitor at higher doses may promptly abrogate cancer cell growth. In this situation, however, it will likely be difficult to discriminate any off-target effects of treatment with the inhibitor from its specific effect on telomerase inhibition.

In this study, we examined the effects of the synthetic telomerase inhibitor MST-312 at higher doses than those in our previous reports^14,16^ and focused on the molecular mechanism for the acute cancer cell growth inhibition induced by high MST-312 concentrations. Exploiting the *in silico* data analysis with our newly developed Telomere Fingerprint Database, we found that MST-312 at high doses works as a dual inhibitor of telomerase and DNA topoisomerase II, an enzyme that regulates the topological state of DNA. Furthermore, using MST-312 as a probe, we demonstrated that both telomere length and lamin A, an inner nuclear membrane protein, are the functional determinants for cancer cell sensitivity to MST-312 and other DNA damaging anticancer agents.

## Results

### MST-312 inhibits human cancer cell growth in mouse xenograft models

**Figure 1:**
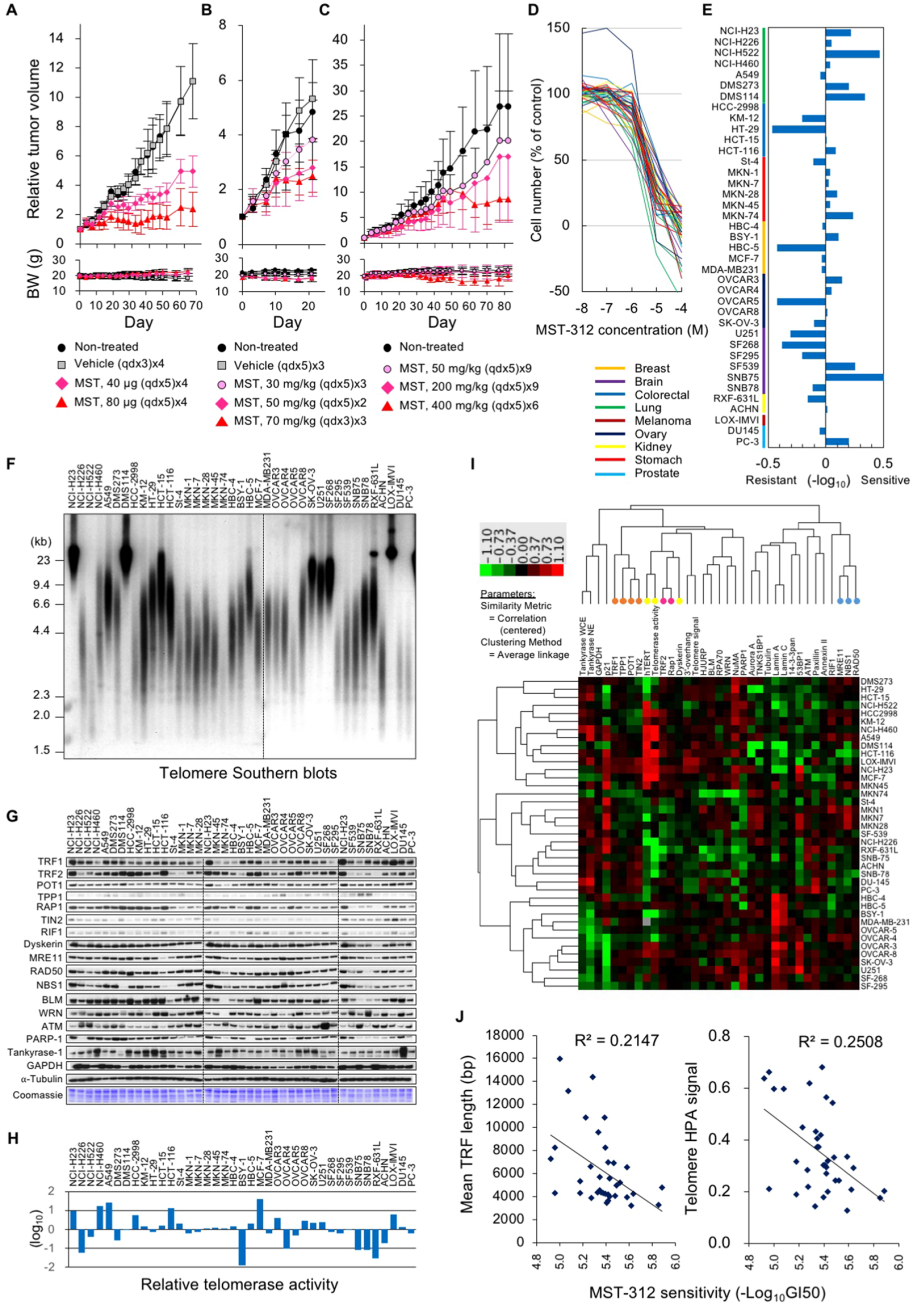
Acute anticancer effect of MST-312 inversely correlates with telomere length of cancer cells. (**A**–**C**) *In vivo* anti-tumour effect of MST-312 in mouse xenograft models. Human breast cancer HBC-4 cells were subcutaneously injected into nude mice. Mice were treated with intratumoural (**A**), intravenous (**B**) or oral administration (**C**) of vehicle or MST-312. *Error bar* indicates standard deviation. *Upper* and *lower graphs* indicate the relative tumour volume and body weight (BW) of the mice, respectively. (**D**) *In vitro* anti-proliferative effect of MST-312 on the JFCR39 panel of 39 human cancer cell lines. Cells were treated with indicated concentrations (molar) of MST-312 for 48 h and then cell numbers were quantitated. (**E**) Fingerprint of MST-312 sensitivity. GI_50_ values of MST-312 quantitated by (**D**) and the average of all cell lines was defined as zero. (**F**) Telomere blot analysis of JFCR39. Genomic DNA was prepared and subjected to Southern blot analysis with the [^32^P]-labelled telomeric probe to detect telomeric restriction fragments (TRFs). Two different blots were derived from the same experiment and were processed in parallel. Their border was indicated by a dotted line. (**G**) Expression of telomere-related proteins in JFCR39. Cell lysates were prepared and subjected to western blot analyses with indicated primary antibodies. For each antibody blot and Coomassie stain, three different blots or gels were derived from the same experiment and were processed in parallel. Their borders were indicated by dotted lines. Each blot/gel contains NCI-H23 cells as a calibration standard. Full-length blots were presented in Supplementary Fig. S4. (**H**) Telomerase activity in JFCR39 cells. Cell lysates were prepared and subjected to TRAP assay. Average telomerase activity of all cell lines was defined as zero. (**I**) Two-dimensional hierarchical cluster analysis of the telomere-related bioparameters. The clustering result was generated by Cluster (ver. 3.0) and Java TreeView (Ver. 1.1.6r4). *Orange dots*: four of six shelterin components (TRF1, POT1, TIN2 and TPP1); *yellow dots*: telomerase components (hTERT and Dyskerin) and telomerase activity; *pink dots*: two shelterin components (TRF2, RAP1); *light blue dots*: MRN complex (MRE11, NBS1 and RAD50). (**J**) Correlation between the cell sensitivity to MST-312 and the length of the mean TRF length (*left*) or telomere signals quantitated by HPA assay (*right*). In these graphs, the super long telomere cells (NCI-H23, DMS114 and LOX-IMVI) were excluded from the calculation of correlation coefficient.

Non-acute cytotoxic doses of the telomerase inhibitor MST-312 shorten telomeres and induce senescence and apoptosis of telomerase-positive human leukaemia and solid tumour cells^14,16^. Meanwhile, MST-312 at higher doses promptly inhibits the proliferation of leukaemia cells^14^. To examine the *in vivo* antitumour efficacy of MST-312, we subcutaneously injected human breast cancer HBC-4 cells into nude mice and treated mice with various doses of MST-312 through various routes. In non-treated and vehicle-treated mice, the tumours grew extensively (Fig. 1A–C). In contrast, intratumoural, intravenous or oral administration of MST-312 at the maximum tolerated or lower doses retarded tumour growth. In mice receiving oral administration of 400 mg/kg MST-312, while a maximum 16.7% body weight loss was observed at day 56, the body weight recovered and returned to levels higher than the original body weight at day 82 (Fig. 1C). Reduction in body-weight was less than 10% during the course of treatments in case of the intratumoural (Fig. 1A) and intravenous (Fig. 1B) MST-312 administration.

### Telomere Fingerprint Database reveals a correlation between shorter telomeres and higher susceptibility to MST-312-induced acute cell growth inhibition

To explore the acute anti-proliferative effects of MST-312, we used a human cancer cell line panel that consists of 39 cell lines derived from various tissues (JFCR39)^28^. Each cell line was treated with various concentrations of MST-312 for 48 h and the anti-proliferative effects were determined (Fig. 1D). The average GI_50_ value of the 39 cell lines was 4.7 μM. The GI_50_ deviation from the average was calculated for each cell line and aligned in a fingerprint (Fig. 1E).

To analyse the relationship between MST-312 sensitivity and telomere status, we quantitated various telomere-related parameters, including the single-stranded (i.e., G-tail/3′-overhang) and the double-stranded telomere length (Fig. 1F); the protein expression for shelterin components (TRF1, TRF2, POT1, TPP1, RAP1, TIN2), DNA damage response factors (RIF1, MRE11, RAD50, NBS1, 53BP1, PARP-1), tankyrase and its binding proteins (TNKS1BP1, NuMA), RecQ helicases (WRN, BLM) and other related proteins (Fig. 1G); telomerase activity (Fig. 1H); and *hTERT* gene expression (Fig. 1I, column 9 from the left). Two-dimensional hierarchical clustering grouped several factors according to their functional relevance (Fig. 1I). For example, components for the MRN complex, MRE11, NBS1 and RAD50 (light blue dots), were classified into the same cluster. In addition, four of six shelterin components, TRF1, POT1, TIN2 and TPP1 (orange dots), were within the same cluster, whereas the other two direct binding components, TRF2 and RAP1 (pink dots), were closely bound. Another larger cluster contained *hTERT*, telomerase activity, and its binding protein dyskerin (yellow dots). These observations suggest that our newly constructed telomere fingerprint database might be able to predict the relative intensity of the physical or functional interaction between various combinations of the fingerprints. There was no significant similarity between the fingerprints of MST-312 and telomere-related parameters. For example, while MST-312 is a telomerase inhibitor, the cell sensitivity to this compound did not correlate with telomerase activity (*r* = −0.094, *P* = 0.582) or *hTERT* mRNA expression (*r* = 0.131, *P* = 0.439) (Supplementary Fig. S1A,B).

When three cell lines, DMS114, NCI-H23 and LOX-IMVI, which possess very long telomeres [more than 20 kb, designated as super long telomere (SLT) type cells] (Fig. 1F), were excluded, we found that acute sensitivity to MST-312 correlated with the length of the double-stranded telomere DNA (Fig. 1J). The cells with shorter telomeres tended to be more susceptible to the acute deleterious effect of MST-312. Furthermore, among the cell lines with very short telomeres (i.e., the mean TRF length is less than 4 kb), there was an inverse correlation between telomerase activity and MST-312 sensitivity (*r* = −0.826, *P* = 0.0415, Supplementary Fig. S1C).

**Figure 2:**
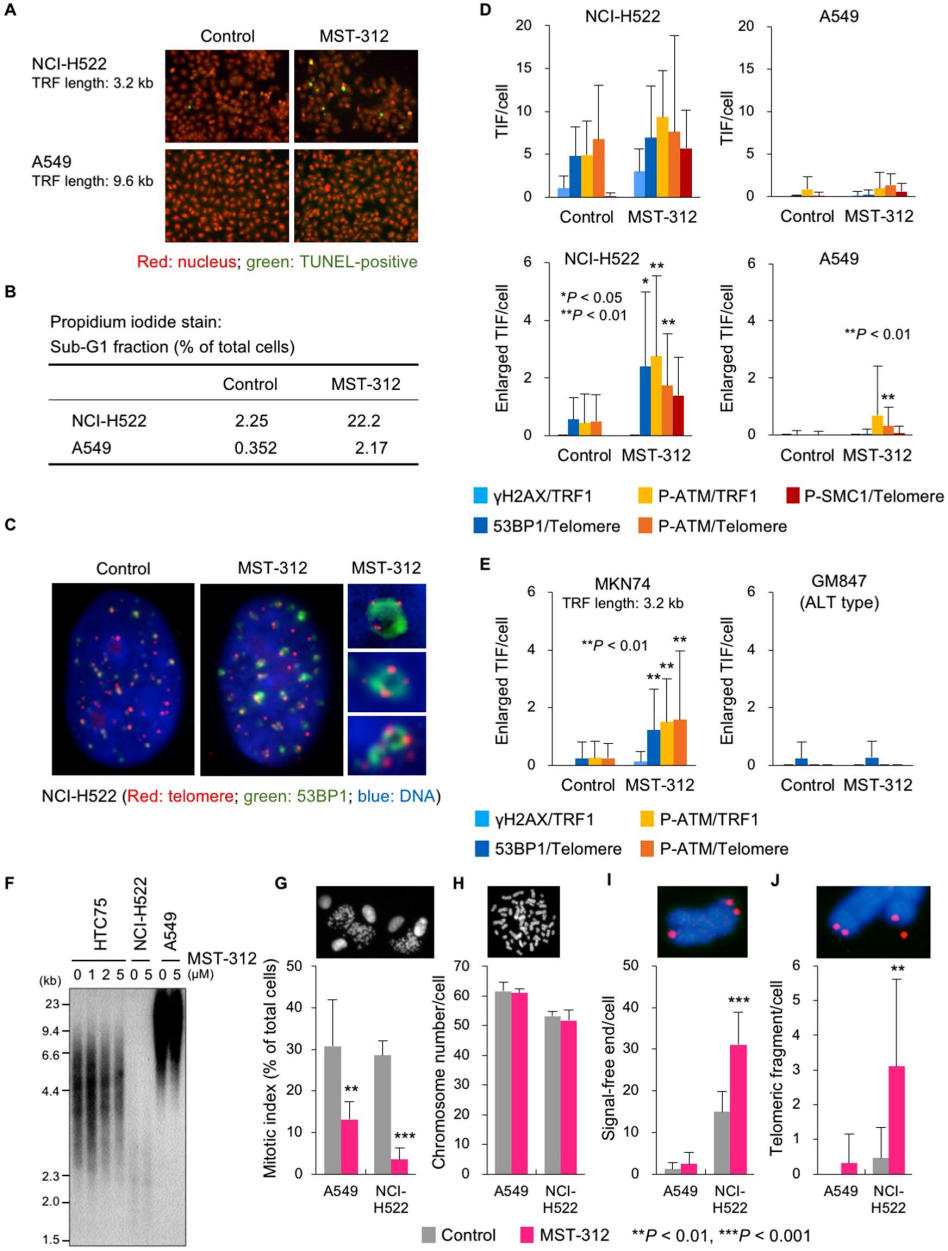
Telomere dysfunction induced by MST-312. (**A**) TUNEL assay of MST-312-treated cells. Human lung cancer NCI-H522 [mean telomeric restriction fragment (TRF) length = 3.2 kb] and A549 (mean TRF length = 9.6 kb) cells were treated with 5 μM MST-312 for 48 h and then subjected to TUNEL assay for detection of apoptotic cells (*green*). (**B**) Quantification of apoptotic cells. Cells were treated as in (**A**) and then stained with propidium iodide; the apoptotic sub-G1 fraction was quantitated by flow cytometry. (**C**) Immuno-FISH analysis of NCI-H522 cells treated with 5 μM MST-312 for 48 h. *Red*: telomere DNA; *green*: 53BP1; *blue*: DAPI. Right panels are magnified views of enlarged telomere dysfunction-induced foci (TIF). (**D**,**E**) Quantitation of TIF. Cells were treated as in (**A**), and telomeres and the indicated proteins were detected by FISH and immunofluorescence staining, respectively. Because the cells with short telomeres gave high background levels of TIF even without MST-312 treatment (**D**, *upper left*), enlarged TIF were quantitated as a hallmark of the MST-312-induced telomeric DNA damage response. *Error bar* indicates standard deviation. *Asterisk* indicates statistical significance in the difference between control and MST-312-treated cells (unpaired two-tailed *t* test). ALT: alternative lengthening of telomeres. (**F**) Telomere southern blot analysis. Cells were treated with indicated doses of MST-312 for 48 h. HTC75 fibrosarcoma cells were analysed as a control because the telomere length fluctuation of this cell line can be detected by southern blot analysis. (**G**) Cells were treated as in (**A**) and mitotic index was quantitated. (**H**–**J**) Cells in (**G**) were further incubated with colcemid. Metaphase spreads of chromosomes were subjected to telomere FISH, and chromosome number (**H**), telomeric signal-free ends (**I**) and telomeric fragments (**J**) were quantitated. *Red*: telomere DNA, *blue*: DAPI stain of chromosome DNA. *Asterisk* indicates statistical significance (vs. control, unpaired two-tailed *t* test).

We further examined the effect of MST-312 on lung cancer NCI-H522 and A549 cells, which retain short and long telomeres with a mean telomeric restriction fragment (TRF) length of 3.2 kb and 9.6 kb, respectively. The GI_50_ values for MST-312 in NCI-H522 and A549 cells were 1.4 μM and 4.6 μM, respectively. Treatment with 5 μM MST-312 for 48 h induced apoptosis of NCI-H522 more frequently than A549 cells (Fig. 2A,B). Consistent with these observations, NCI-H522 cells showed enlarged telomere dysfunction-induced foci (TIF) of DNA damage response factors, such as γH2AX, 53BP1, phosphorylated ATM kinase and phosphorylated SMC1, upon treatment with 5 μM MST-312, whereas A549 did not (Fig. 2C,D). MST-312-induced enlarged TIF formation was also observed in another cell line with short telomeres, stomach MKN74 cells (mean TRF length: 3.2 kb), but not in telomerase-independent immortalised, alternative lengthening of telomere (ALT) type GM847 cells, which possess a homologous recombination-based telomere maintenance mechanism^29^ (Fig. 2E). Under these short term treatment conditions, telomere shortening was not detectable by Southern blot analysis (Fig. 2F). Furthermore, MST-312 reduced the mitotic index in A549 and NCI-H522 cells (Fig. 2G) without affecting chromosome numbers (Fig. 2H) and caused telomeric signal-free ends (Fig. 2I) and telomere fragmentation (Fig. 2G) only in NCI-H522 cells. These observations indicate that MST-312 treatment at high doses for 48 h causes telomere dysfunction in cancer cells with short telomeres.

### MST-312 induces DNA double-strand breaks in the drug-sensitive cancer cells

**Figure 3:**
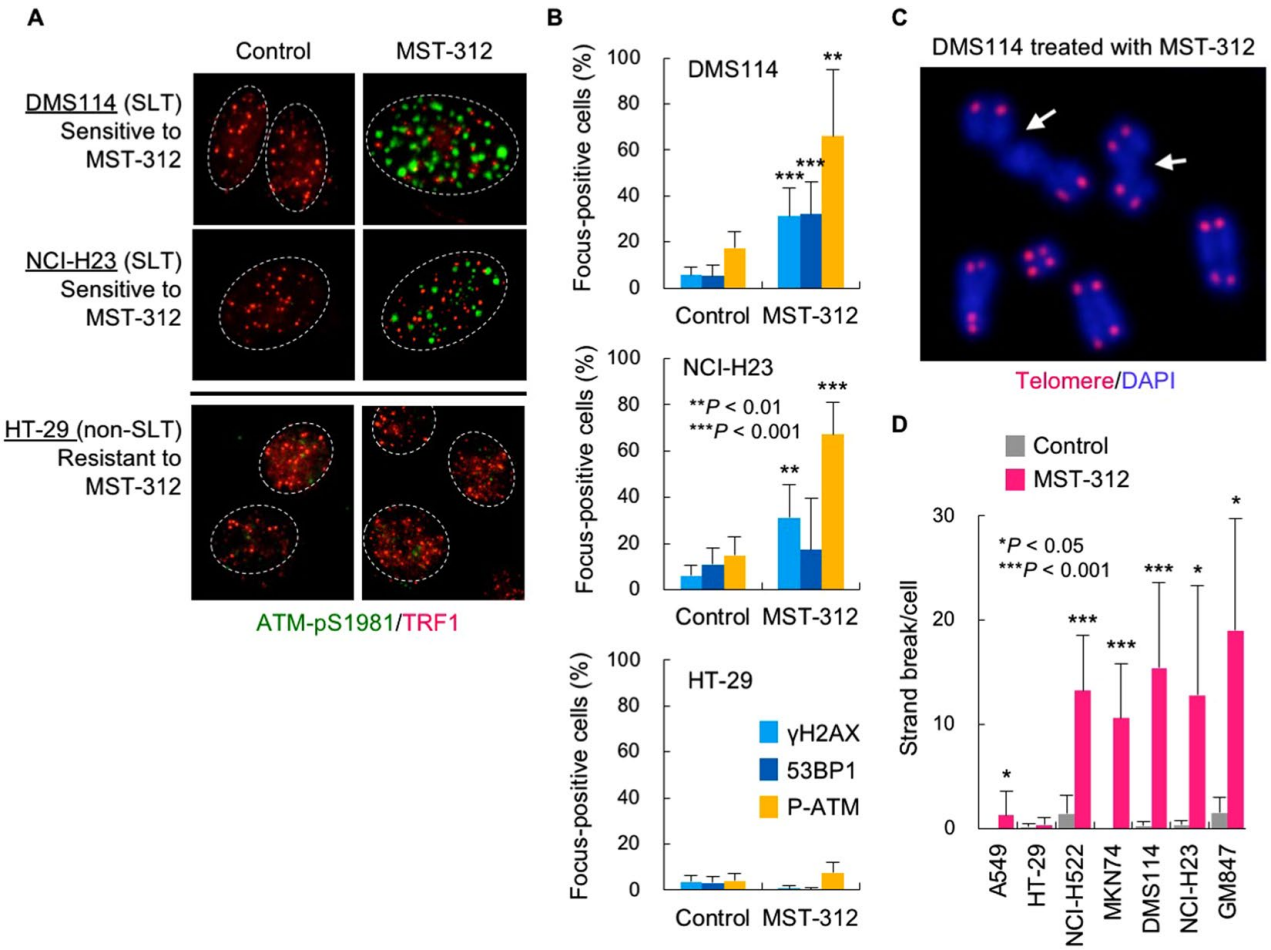
MST-312 induces DNA double-strand breaks in a telomerase-independent manner. (**A**) Non-telomeric DNA damage response induced by MST-312. MST-312-sensitive NCI-H23 [super long telomere (SLT) type: mean TRF length = 22 kb] and DMS114 cells (SLT type: mean TRF length = 23 kb) and MST-312-resistant HT-29 cells (mean TRF length = 7.3 kb) were treated with 5 μM MST-312 for 48 h. Cells were subjected to indirect immunofluorescence staining with anti-phospho-ATM (p1981) (*green*) and anti-TRF1 (*red*) antibodies. (**B**) Quantitative graphs of non-telomeric DNA damage foci in NCI-H23, DMS114 and HT-29 cells. Cells were treated as in (**A**) and immunofluorescence staining was performed with the indicated primary antibodies. Cells with more than four foci were counted as positive. (**C**) DNA strand breaks induced by MST-312. DMS114 cells were treated as in (**A**) and further incubated with colcemid for 5 h. Metaphase spreads of chromosomes were subjected to telomere FISH analysis. *Red*: telomere DNA; *blue*: DAPI stain of chromosome DNA; *arrow*: chromatid break. (**D**) Quantitation of DNA strand breaks in MST-312-treated cells. Cells were treated as in (**A**) and subjected to telomere FISH analysis. GM847 is a telomerase-independent ALT type cell line. *Error bar* indicates standard deviation. *Asterisk* indicates statistical significance (vs. control, unpaired two-tailed *t* test).

Having confirmed telomere dysfunction upon MST-312 treatment of cells that have short telomeres, we next examined the effect of MST-312 on two SLT type cell lines, DMS114 and NCI-H23. We found that both cell lines were sensitive to MST-312 (GI_50_ values: 2.5 μM and 1.9 μM, respectively) even though these cells possess very long telomeres (mean TRF length: > 20 kb). As shown in Fig. 3A,B, MST-312 treatment of these cells caused DNA damage foci that were not colocalised with telomeres. MST-312-resistant colorectal cancer HT-29 cells (GI_50_: 12 μM; mean TRF length: 7.3 kb) did not exhibit foci upon treatment. Consistent with these observations, metaphase spreads of chromosomes showed that MST-312 caused DNA double-strand breaks in MST-312-sensitive cells (NCI-H522, MKN74, DMS114 and NCI-H23) but not in MST-312-resistant cells (A549 and HT-29) (Fig. 3C,D). Furthermore, the telomerase negative (ALT type) GM847 cells also exhibited DNA double-strand breaks in response to MST-312 (Fig. 3D). These results suggest that MST-312 at a higher dose (e.g., 5 μM) than that for telomerase inhibition (~1 μM) can induce DNA double-strand breaks in a telomerase-independent manner.

Then, we next knocked down hTERT in A549 and NCI-H522 cells and evaluated their MST-312 sensitivities. In the absence of MST-312, siRNA-mediated knockdown of hTERT reduced the growth of A549 and NCI-H522 cells to 77% and 58%, respectively, of the non-silencing control siRNA-treated cells. It has been reported that knockdown, instead of enzymatic inhibition, of telomerase exerts such an acute anticancer effect in an enzyme activity-independent manner^30^. We confirmed that this siRNA had no effect on the growth of telomerase-independent GM847 cells, excluding the possibility of an off-target effect (Kyotaro Hirashima and HS, unpublished observation). Under these hTERT-depleted conditions, MST-312 further inhibited the cell growth in a dose-dependent manner (Supplementary Fig. S1D,E).

### COMPARE analysis of chemical fingerprints identifies MST-312 as a DNA topoisomerase II inhibitor

**Figure 4:**
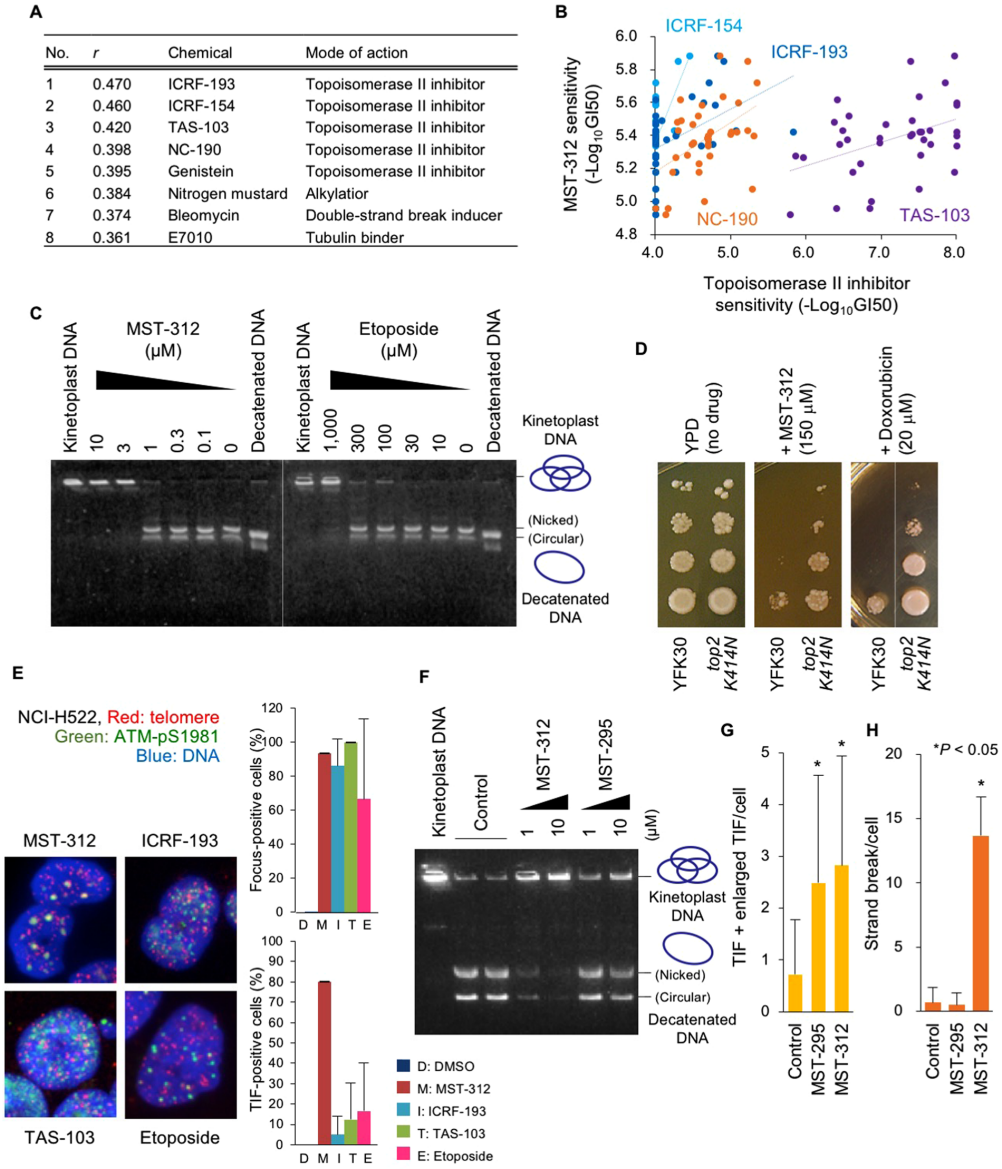
MST-312 inhibits DNA topoisomerase II. (**A**) COMPARE analysis of MST-312 with anticancer compounds. Similarities between chemical fingerprints of MST-312 and anticancer compounds in the JFCR39 cancer cell line panel were evaluated. (**B**) Correlation between sensitivities to MST-312 and DNA topoisomerase II (Topo II) inhibitors ICRF-193 (*blue*, R² = 0.2101), ICRF-154 (*dark blue*, R² = 0.24561), NC-190 (*orange*, R² = 0.22231) and TAS-103 (*purple*, R² = 0.17742). (**C**) *In vitro* Topo II enzyme assay. Kinetoplast DNA was incubated with Topo II in the presence of the indicated concentrations of the compounds and subjected to agarose gel electrophoresis. Decatenated DNA is a marker of Topo II reaction. Border of two different gels, which were derived from the same experiment and processed in parallel, was indicated by a dotted line. (**D**) Spotting assay of 10-fold serial dilution of the yeast parental strain YFK30 and *top2 K414N* mutant. The 10-fold dilutions of log-phase cells were spotted onto YPD plates containing the indicated concentrations of MST-312 or the positive control doxorubicin and then cells were cultivated at 30 °C. In the right panel, border of two different parts of the same plate was indicated by a dotted line. (**E**) Telomere immuno-FISH analysis. NCI-H522 cells were treated with 5 μM MST-312 or Topo II inhibitors (150 μM ICRF-193, 0.2 μM TAS-103 or 8.5 μM etoposide) for 48 h and then subjected to telomere immuno-FISH analysis. *Left*: representative photos. *Red*: telomere DNA; *green*: phospho-ATM (p1981); *blue*: DAPI stain of DNA. *Right*: quantitative graphs of phospho-ATM (p1981) and TIF-positive cells. Cells with more than four foci were counted as positive. (**F**) Effect of MST-295 on Topo II enzyme activity. (**G**) TIF induced by MST-295 in NCI-H522 cells. Cells were treated with 5 μM MST-295 or MST-312 for 48 h and then subjected to telomere immuno-FISH. Numbers of TIF and enlarged TIF were quantitated. (**H**) Effect of MST-295 on DNA double strand breaks in DMS114 cells. Cells were treated as in (**G**) and metaphase spreads of chromosomes were analysed for DNA double strand breaks. *Error bar* indicates standard deviation. *Asterisk* indicates statistical significance (Tukey-Kramer test).

The ability of MST-312 to induce DNA damage in GM847 cells suggests that MST-312 has another molecular target in addition to telomerase. To identify potential targets, we performed COMPARE analysis^12,31^ using chemical fingerprints of MST-312 and various anticancer compounds. We found that MST-312 has a similar fingerprint to fingerprints of DNA topoisomerase II inhibitors, such as ICRF-193, ICRF-154, TAS-103 and NC190 (Fig. 4A,B). This result prompted us to perform DNA decatenation assays, which monitor DNA topoisomerase II activity to decatenate the kinetoplast DNA. As shown in Fig. 4C, MST-312 inhibited DNA topoisomerase II with an IC_50_ value of 2 μM, which was three-fold higher than the IC_50_ value for telomerase inhibition^14^. Downregulation of DNA topoisomerase II is a well-established mechanism for resistance to DNA topoisomerase II inhibitors^32^. MST-312 at 150 μM inhibited the growth of *Saccharomyces cerevisiae* YFK30, a drug-hypersensitive strain^33^ (Fig. 4D). In contrast, the DNA topoisomerase II-mutant in the YFK30 background, *top2*, *K414N*, was resistant to the growth inhibitory effect of MST-312. In the control treatment, this mutant strain was also resistant to doxorubicin, another DNA topoisomerase II inhibitor. iFISH analysis revealed that DNA topoisomerase II inhibitors, such as ICRF-193, TAS-103 and etoposide, induced non-telomeric DNA damage foci that were distinct from the TIF induced by MST-312 in NCI-H522 cells (Fig. 4E, *left panel*). While the percentages of the ATM-pS1981 DNA damage focus-positive cells were comparable between the drug-treated cells, the percentages of the TIF-positive cells were significantly higher in MST-312-treated cells than in other drug-treated cells (Fig. 4E, *right panel*). MST-295, a telomerase inhibitor with telomerase inhibiting activity comparable to MST-312^14^, only marginally inhibited DNA topoisomerase II (Fig. 4F). MST-295 induced TIF and enlarged TIF in NCI-H522 cells, indicating telomerase inhibition (Fig. 4G). However, MST-295 did not induce DNA double-stranded breaks (Fig. 4H). These observations indicate that MST-312 used at 5 μM and higher doses works as a dual inhibitor for telomerase and DNA topoisomerase II and induces telomere dysfunction in cancer cells with short telomeres and non-telomeric DNA damage in cancer cells that do not necessarily have short telomeres.

### Lamin A is a functional determinant for MST-312-induced DNA double-strand breaks

**Figure 5:**
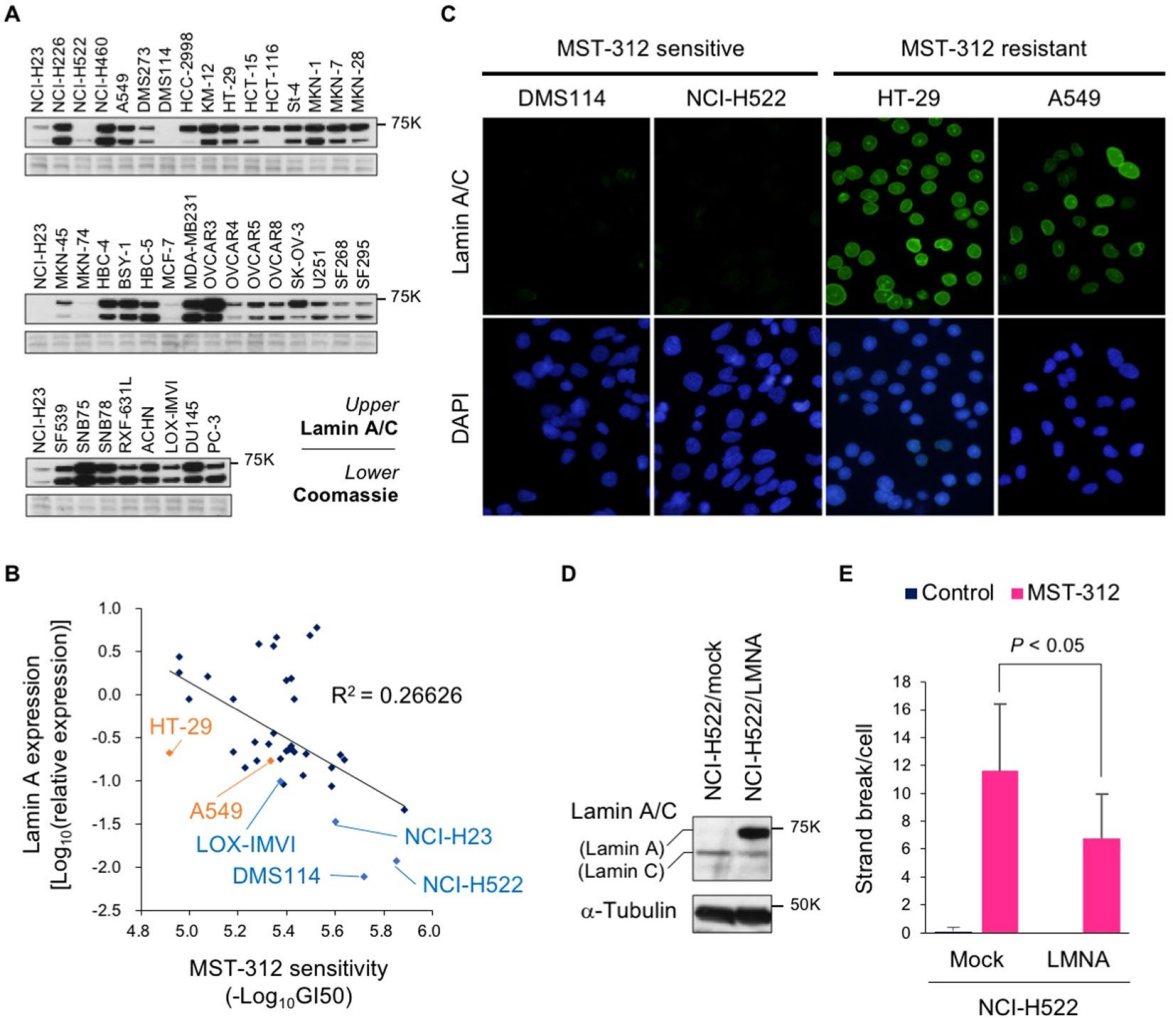
Lamin A prevents MST-312-induced DNA double strand breaks. (**A**) Western blot analysis of lamin A/C in the JFCR39 cancer cell line panel. Coomassie brilliant blue gel staining is shown to confirm equal loading. Three different blots or gels were derived from the same experiment and were processed in parallel. Each blot/gel contains NCI-H23 cells as a calibration standard. Full-length blots/gels were presented in Supplementary Fig. S5. (**B**) Correlation between MST-312 sensitivity and lamin A protein expression level in JFCR39. Representative cell lines are shown in *orange* and *blue*. GI_50_ values were determined by the cell growth inhibition curve of 48-h treated cells in Fig. 1D. (**C**) Indirect immunofluorescence staining with anti-lamin A/C antibody (*green*). MST-312-sensitive [NCI-H522 (short telomeres) and DMS114 cells (super long telomeres)] and MST-312-resistant cells (HT-29, A549) are shown. DNA was counterstained with DAPI (*blue*). (**D**) Ectopic expression of lamin A in NCI-H522 cells. Cells were infected with retrovirus for expression of lamin A (LMNA) exogene and selected by hygromycin. The resulting cells, NCI-H522/mock and NCI-H522/LMNA, were subjected to western blot analysis. α-tubulin served as loading control. Full-length blots were presented in Supplementary Fig. S5. (**E**) Cells in (**D**) were treated with 5 μM MST-312 for 48 h and further incubated with colcemid. Metaphase spreads of chromosomes were analysed for DNA double strand breaks. *Error bar* indicates standard deviation. Statistical significance was evaluated by Tukey-Kramer test.

As described above, MST-312 did not induce the DNA damage response in some cancer cell lines, such as A549 and HT-29 cells. To identify the functional determinant for the susceptibility to MST-312-induced non-telomeric DNA damage, we performed BIO-COMPARE analysis^34^ and examined the similarity between the chemical fingerprint of MST-312 sensitivity and biological fingerprints of protein expression. We found that the expression levels for lamin A, an inner nuclear membrane protein, inversely correlated with MST-312 sensitivity with the highest correlation (*r* = −0.518, *P* = 0.0006) (Fig. 5A,B). Meanwhile, there was a weak correlation between expression levels of lamin C, a splicing valiant of *LMNA* gene, and MST-312 sensitivity (*r* = −0.389, *P* = 0.0137) (Supplementary Fig. S2A). Importantly, SLT-type DMS114, NCI-H23 and LOX-IMVI cells, which retain very long telomeres and are sensitive to MST-312, expressed the lowest levels of lamin A protein. NCI-H522 cells, which retain very short telomeres and are sensitive to MST-312, also expressed very low levels of lamin A. MST-312-resistant cells, such as A549 and HT-29 cells, expressed high levels of lamin A. These expression patterns were confirmed by indirect immunofluorescent staining for lamin A (Fig. 5C).

To examine whether lamin A is functionally involved in the susceptibility to MST-312-induced DNA damage, NCI-H522 cells were infected with retrovirus containing lamin A (*LMNA*) cDNA and selected with hygromycin (Fig. 5D). The resulting NCI-H522/LMNA cells were treated with 5 μM MST-312 for 48 h and DNA double-stranded breaks were quantitated. As shown in Fig. 5E, lamin A overexpression in NCI-H522 cells reduced the number of MST-312-induced double-stranded breaks. By contrast, lamin C overexpression did not affect the formation of DNA damage foci upon treatment with MST-312 (Supplementary Fig. S2B). These results suggest that lamin A expression is a functional determinant of the sensitivity to the DNA damage acutely induced by higher doses of MST-312.

### Telomere elongation and lamin A expression alleviate MST-312-induced apoptosis

**Figure 6:**
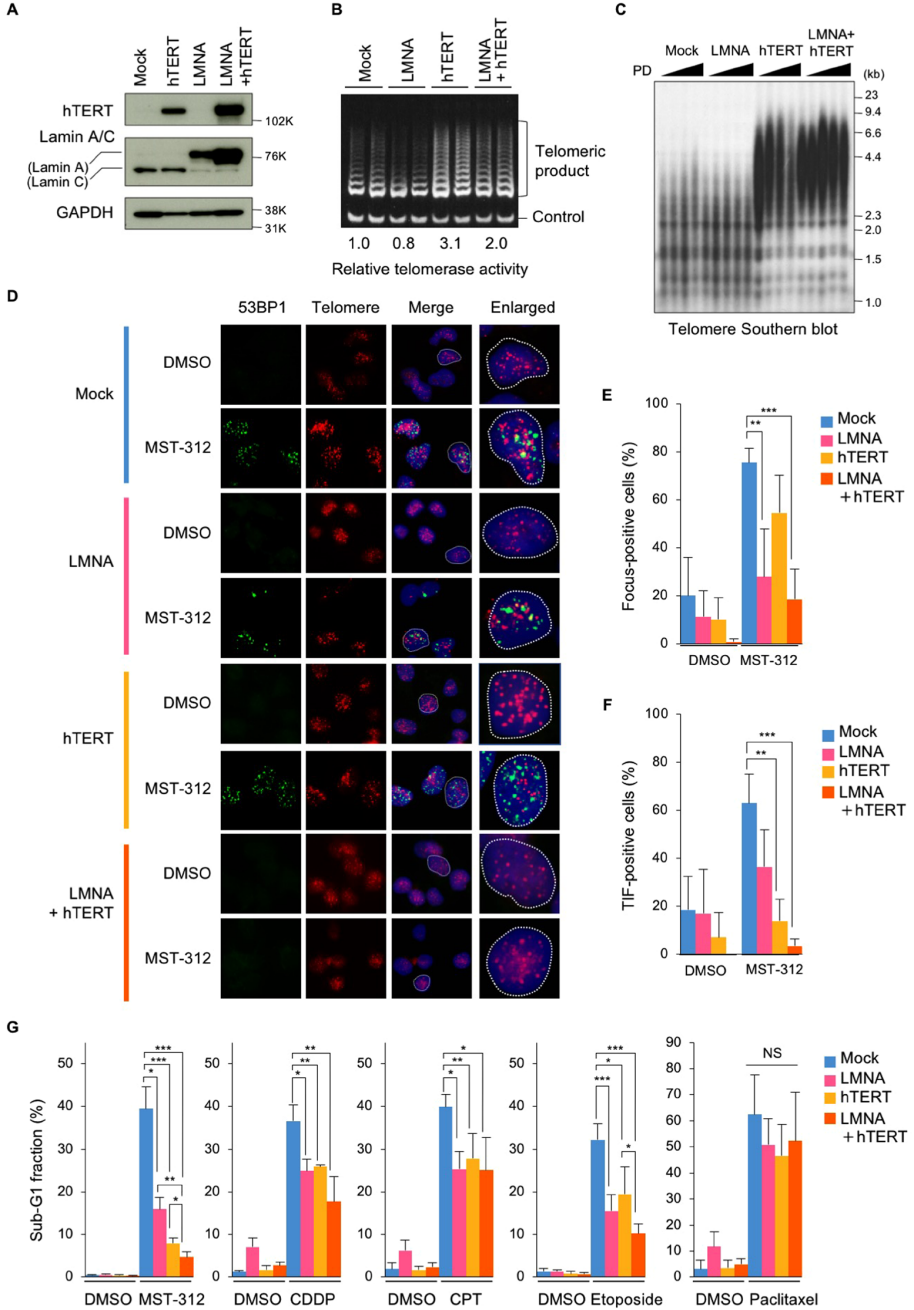
Telomere elongation and lamin A expression confers resistance to MST-312. (**A**,**B**) Telomere elongation and lamin A expression in NCI-H522 cells. Cells were infected with hTERT and/or LMNA expressing retrovirus. Cells were subjected to western blot analysis for detection of hTERT and lamin A expression (**A**) and TRAP assay for detection of telomerase activity (**B**). For (**A**) full-length blots were presented in Supplementary Fig. S5. (**C**) Telomere southern blot analysis. Genomic DNA was prepared and subjected to southern blot analysis with [^32^P]-labelled telomeric probe. (**D**) Telomere immuno-FISH analysis. Cells were treated with 5 μM MST-312 for 48 h and subjected to immuno-FISH analysis. *Green*: 53BP1; *red*: telomere DNA; *blue*: DAPI stain of DNA. (**E**) Quantitation of DNA damage foci in (**D**). Cells with more than four 53BP1 foci were counted as positive. (**F**) Quantitation of telomeric DNA damage (TIF and enlarged TIF) in (**D**). Cells with more than four TIF were counted as positive. (**G**) Apoptosis induced by MST-312 and anticancer drugs. Cells were treated with 5 μM MST-312, 3.3 μM cisplatin (CDDP), 10 nM camptothecin (CPT), 1.6 μM etoposide or 5 nM paclitaxel for 96 h. Cells were stained with propidium iodide and the apoptotic sub-G1 fraction of the cell cycle was quantitated by flow cytometry. *Error bar* indicates standard deviation of four (**E**,**F**) or at least three (**G**) experiments. Statistical significance was evaluated by Tukey-Kramer test. *P < 0.05, **P < 0.01, ***P < 0.001. NS: not significant.

Because cancer cells with shorter telomeres tended to be more sensitive to MST-312 than cells with longer telomeres (Fig. 1J), we next transfected NCI-H522 or NCI-H522/LMNA cells with an expression vector encoding the telomerase catalytic subunit, hTERT, and upregulated the telomerase activity in the cells (Fig. 6A,B). The resulting NCI-H522/hTERT and NCI-H522/LMNA + hTERT cells exhibited elongated telomeres (Fig. 6C). Immuno-FISH analysis revealed that the telomeric and non-telomeric DNA damage induced by MST-312 were reduced by lamin A and hTERT expression, respectively, and simultaneous overexpression of the two proteins eliminated the DNA damage foci in the nuclei (Fig. 6D–F). Consistent with these observations, expression of either lamin A or hTERT protein reduced the levels of apoptosis induced by MST-312 in NCI-H522 cells, and simultaneous overexpression of both proteins further inhibited apoptosis (Fig. 6G). Lamin A and hTERT also reduced the rates of apoptosis induced by DNA damaging agents, such as cisplatin, camptothecin and etoposide, but not by the anti-microtubule agent paclitaxel (Fig. 6G). These observations indicate that telomere length and lamin A expression are the critical determinants for telomeric and non-telomeric DNA damaging agents.

Lamin A overexpression not only reduced lamin C expression but also slightly reduced telomerase activity (Fig. 6A,B), suggesting the possibility that lamin C expression plays a supportive role for telomerase activity. Then, we reconstituted lamin C expression in NCI-H522/mock and NCI-H522/LMNA cells and quantified the telomerase activity (Supplementary Fig. S3). The results showed that lamin C expression does not enhance telomerase activity in NCI-H522/mock cells (lanes 1/2 and 5/6). Meanwhile, a slight downregulation of telomerase activity in NCI-H522/LMNA cells was marginally rescued by lamin C expression (lanes 3/4 and 7/8). These observations suggest that lamin C supports telomerase activity to some extent, but it is not sufficient to rescue the MST-312-induced double-stranded breaks (Supplementary Fig. S2).

## Discussion

In cultured cancer cells, telomerase inhibitors can allow for telomerase shortening, restoring the end replication problem, and inducing eventual telomere crisis^14^. This pharmacological effect is achieved by using intentionally low doses of telomerase inhibitors that would not induce acute cytotoxicity. Here we abandoned the classical idea that telomerase inhibitors should be evaluated at such low doses. As shown in Fig. 1D, treatment with the telomerase inhibitor MST-312 at higher doses exhibited acute anti-proliferative effects. Upon investigating the molecular mechanism for this rapid anticancer effect of MST-312, we found that the compound, when used at high doses, is a dual inhibitor of telomerase and DNA topoisomerase II and can induce telomeric and non-telomeric DNA damage.

Exploiting the telomere fingerprints of 39 human cancer cell lines, we found that cancer cell lines with shorter telomeres are more sensitive to the acute deleterious effects of MST-312. This result makes sense because shorter telomeres would allow fewer numbers of cell division to occur and promptly cause telomere crisis in the absence of telomerase activity. In fact, telomere elongation by overexpression of telomerase (this study) or tankyrase^16^, a positive regulator of telomerase, confers resistance to MST-312. Consistent with these observations, a preclinical study suggested that telomere length could be a predictive biomarker of the telomerase inhibitor imetelstat^35^. Furthermore, a randomised phase II study of imetelstat has demonstrated a trend toward longer median progression-free survival and overall survival in imetelstat-treated patients with non-small cell lung cancer with short telomeres^19^. Intriguingly, cancer cells often maintain shorter telomeres than those in cells in surrounding normal tissues^36,37^, and short telomeres are associated with a less-differentiated status of tumours *in vivo*^38,39^. In this sense, telomerase inhibitors may be suitable for targeting such malignant tumours.

We cannot precisely quantitate the level of telomerase activity in MST-312-treated cells. Because MST-312 is a reversible telomerase inhibitor, it is washed out during the course of lysate preparation from the drug-treated cells. Accordingly, telomerase activity is restored in the cell lysate^14^. For these reasons, it is difficult to evaluate the relationship between telomerase activity in MST-312-treated cells and their drug sensitivities. Telomerase can be also inhibited by siRNA-mediated knockdown. However, effects of hTERT knockdown and telomerase inhibition are not necessarily equivalent each other and, therefore, it was difficult by the knockdown approach to check if MST-312 sensitivity is partially mediated by the inhibition of telomerase activity. Still, however, MST-312 resistance by hTERT overexpression (Fig. 6) would support that this drug sensitivity is partially mediated by telomerase inhibition.

We have also demonstrated that the expression levels of lamin A protein are inversely correlated with the susceptibility to apoptosis by treatment with MST-312 and DNA damaging anticancer drugs but not by paclitaxel. Lamin A is one of the nuclear matrix proteins and interacts with several proteins, such as LAPs 1A, 1B^40^, emerin^41^, retinoblastoma transcription factor pRB^42^ and myne-1^43^. Among these proteins, both emerin and pRB associate with DNA, indicating that lamin A interacts with several DNA-binding proteins and plays important roles in the nucleus. In fact, loss of lamin A function increases the chromatin motion^44–47^. Mutations in the LMNA gene, which encodes the two nuclear lamins, lamin A and C, produced by alternative splicing, cause a wide range of diseases called laminopathies^48,49^. With regard to the association between senescence and lamin A function, much can be learned about the premature aging syndrome Hutchinson-Gilford progeria syndrome (HGPS)^50,51^. In cases with a splicing mutation of LMNA, the mutant lamin A protein, progerin, accumulates in HGPS patient cells. Progerin has a 50-amino-acid internal deletion and lacks the proteolytic cleavage site necessary to remove the carboxy-terminal 18 amino acids to generate mature lamin A^50,51^. Progerin acts as a dominant negative mutant of lamin A and causes dysregulation of the DNA damage response^48,49,52–55^. HGPS patient-derived cells are highly sensitive to ionising radiation-induced DNA damage, and ectopic expression of progerin mimics this phenotype^54^. At the molecular level, progerin traps DNA-dependent protein kinase, a critical protein in double-strand break repair^56^. Loss of wild-type lamin A can also result in perturbed non-homologous end joining and homologous recombination^57^. Our BIO-COMPARE analysis of the JFCR39 panel showed that the expression level of lamin A protein inversely correlates with the sensitivity to various DNA damaging drugs (YM, TY and HS, unpublished observations). These observations support the functional importance of lamin A for proper DNA damage response and repair.

hTERT-mediated immortalisation of HGPS cells improves nuclear morphology, decreases progerin protein levels and diminishes the number of persisting 53BP1 foci, alleviating the dysregulation in the DNA damage response and repair machinery^54^. Furthermore, loss of lamin A alters the nuclear distribution and heterochromatin status of telomeres^58^. These observations suggest a functional linkage between telomeres and lamin A. Consistent with these data, we found that lamin A overexpression in NCI-H522 cells repressed the telomeric DNA damage induced by MST-312 (Fig. 6F).

Our chemical fingerprinting analysis demonstrates that the MST-312 sensitivity profile at the acute phase is similar to the profiles of DNA topoisomerase II inhibitors, especially ICRF-193. One study showed that ICRF-193 preferentially attacks telomeres in a telomeric protein TRF2-dependent manner^59^. However, we found that ICRF-193 or other DNA topoisomerase II inhibitors, which have no telomerase inhibitory activities, induced less telomeric DNA damage than MST-312 (Fig. 4E). Thus, the mixed phenotype of the telomeric and non-telomeric DNA damage would be caused by the dual inhibition of telomerase and DNA topoisomerase II by MST-312 treatment in NCI-H522 cells. Inhibitors with multiple nuclear target, as demonstrated in this study, may suggest new treatment strategies against cancer.

## Materials and Methods

### Chemicals

MST-312 and MST-295 were synthesised as described previously^14^. ICRF-193 and ICRF-154 were provided by Zenyaku Kogyo Co., Ltd. (Tokyo, Japan). TAS-103 was provided by Taiho Pharmaceutical Co., Ltd. (Tokyo, Japan). Etoposide and cisplatin were purchased from Cosmo Bio Co., Ltd. (Tokyo, Japan). Camptothecin, doxorubicin and paclitaxel were obtained from Sigma-Aldrich (St. Louis, MO, USA).

### Cell culture, cell growth and apoptosis assays

The JFCR39 human cancer cell line panel was described previously^60^. Cells were grown in Dulbecco’s modified Eagle’s medium supplemented with 10% heat-inactivated calf serum and 100 μg/ml of kanamycin at 37 °C in a humidified atmosphere of 5% CO_2_. Cell growth inhibition was assessed by sulforhodamine B assay^61^, the CellTiter 96 AQueous One Solution cell proliferation assay kit (Promega, Fitchburg, WI, USA) or MTT assay^62^. To predict molecular target of MST-312, COMPARE analysis using JFCR39 panel was performed as described previously^12,31^. Terminal deoxynucleotidyl transferase-mediated dUTP nick-end labelling (TUNEL) assay for detection of apoptotic cells was performed using the ApoAlert DNA Fragmentation Assay Kit (Takara Bio, Shiga, Japan) according to the manufacturer’s instructions. Quantitation of the sub-G1 fractions was performed by propidium iodide staining followed by flow cytometry, as described previously^16^.

### *In vivo* xenografts

Human breast cancer HBC-4 cells were implanted subcutaneously in the right flank region of 9-week-old BALB/cAJcl-nu nude mice (Charles River Laboratories Japan, Kanagawa, Japan). Experiments were started when tumours reached 50–150 mm^3^ as measured with callipers (day 0). MST-312 was administered by either intratumoural injection at 40 or 80 μg/day; intravenous injection at 30, 50 or 70 mg/kg/day; or orally at 50, 200 or 400 mg/kg/day. In the intratumoural injection experiment, vehicle group was administrated three times of once daily (qd) per week for four weeks and 40 and 80 μg/day groups were administrated five times of qd per week for four weeks. In the intravenous injection experiment, vehicle and 30 mg/kg groups were administrated five times of qd per week for three weeks, 50 mg/kg group was administrated five times of qd per week for two weeks and 70 mg/kg group was three times of qd per week for three weeks. In the orally administrated group, 50 and 200 mg/kg groups were administrated five times of qd per week for nine weeks and 400 mg/kg group was administrated five times of qd per week for 6 weeks. Control mice (n = 5–6) received the same volume of saline as experimental mice (n = 5–6 per group). Tumour growth was monitored. The length (L) and width (W) of the tumours were measured, and the tumour volume (TV) was calculated as TV = LW^2^/2. All animal procedures were performed in the animal experiment room of the Japanese Foundation for Cancer Research (JFCR) using protocols approved by the JFCR Animal Care and Use Committee.

### Western blot analysis

Whole cell and nuclear extracts were prepared as described previously^63^. Western blot analysis was performed as described^64^ with the following primary antibodies: anti-TRF1 (#5745, 0.5 μg/ml)^63^, anti-TRF2 (4A794, 2.5 μg/ml, Novus Biologicals, Littleton, CO, USA), anti-POT1 (#978, 1:1,000, provided by Dr. Titia de Lange), anti-TIN2 (1:1,500, provided by Dr. Zhou Songyang), anti-TPP1 (#467, 1:1,000, provided by Zhou Songyang), anti-RAP1 (1:1,000, provided by Zhou Songyang), anti-RIF1 (#1060, 1:1,000, provided by Titia de Lange), anti-dyskerin (H-300, 2 μg/ml, Santa Cruz Biotechnology, Dallas, TX, USA), anti-MRE11 (12D7, 1:500, GeneTex, Irvine, CA, USA), anti-RAD50 (13B3, 1:1,000, GeneTex), anti-NBS1 (#3002, 1:1,000, Cell Signaling Technology, Danvers, MA, USA), anti-BLM (ab476, 1:2,000, Abcam, Cambridge, UK), anti-WRN (ab200, 1:2,000, Abcam), anti-ATM (2C1, 2 μg/ml, Santa Cruz Biotechnology), anti-PARP-1 (C2-10, 1:2,000, Pharmingen, Franklin Lakes, NJ, USA), anti-tankyrase-1 (H-350, 2 μg/ml, Santa Cruz Biotechnology), anti-glyceraldehyde-3-phosphate dehydrogenase (GAPDH; RDI-TRK5G4-6C5, 0.5 μg/ml, Research Diagnostics, Flanders, NJ, USA), anti-α-tubulin (B-5-1-2, 1:1,000, Sigma), anti-lamin A/C (636, 2 μg/ml, Santa Cruz Biotechnology) and anti-hTERT (1531-1/Y182, 1:1,000, Abcam). Images were processed with Photoshop CS5 (Adobe).

### Telomere and telomerase assays

Genomic DNA was isolated and telomere restriction fragments (TRFs) were detected by Southern blot analysis as previously described^38^. The mean length of TRFs was determined with an Atto densitoscan analyser (Tokyo, Japan). All procedures were performed in accordance with the institutional guideline and regulation under approval by the JFCR Radiation Safety Committee. Telomeric DNA signals and the telomeric 3′-overhang were quantitated by HPA assay as described previously^65^. TRAP assay for estimation of telomerase activity was performed as previously described^38^.

### RT-PCR

Total RNAs were prepared using the RNeasy Mini kit (Qiagen, Venlo, Netherlands), and *hTERT* expression was monitored by RT-PCR using Ready-To-Go beads (Amersham Pharmacia, Piscataway, NJ, USA) and a pair of primers, 5′-TTGGTGCACACCGTCTGGAGG-3′ and 5′-CTGGAGGTGCAGAGCGACTAC-3′. The PCR conditions were 29 temperature cycles of 94 °C for 45 sec, 60 °C for 45 sec, and 72 °C for 90 sec. Signals were detected by agarose gel electrophoresis and normalised by *β-microglobulin* expression, which was detected by primers 5′-ACCCCCACTGAAAAAGATGA-3′ and 5′-ATATTCAAACCTCCATGATG-3′, under conditions of 24 temperature cycles of 94 °C for 30 sec, 55 °C for 1 min and 72 °C for 2 min. In pilot studies, we determined the quantitative range of the reaction by modifying the number of temperature cycle.

### Immunofluorescence and immuno-FISH (iFISH) assays

Immunofluorescence staining and iFISH assays were performed as described^64^. The following primary antibodies were used: anti-human TRF1 (5747, 1 μg/ml)^63^, anti-ATM pS1981 (200-301-400 or 600-401-400, 7.5 μg/ml, Rockland Immunochemicals, Limerick, PA, USA), anti-53BP1 (#4937, 1:100, Cell Signaling Technology), anti-SMC1 pSer966 (BL311, 2 μg/ml, Bethyl Laboratories, Montgomery, TX, USA) and anti-γH2AX (JBW301, 2 μg/ml, BD Biosciences, San Jose, CA, USA). For telomere PNA FISH, cells were treated with 0.25 μg/ml colcemid for 3 h, trypsinised and swollen in 0.6% sodium citrate for 30 min at 37 °C. Metaphase spreads were prepared on slide glass and telomere PNA FISH was performed as described^16^. Images were processed with Photoshop CS5 (Adobe).

### DNA topoisomerase II assay

*In vitro* DNA topoisomerase II assay was performed using the Topo II Assay Kit (TopoGEN, Buena Vista, CO, USA) according to the manufacturer’s instructions. In brief, kinetoplast DNA was incubated with 2 units of DNA topoisomerase II for 1 h at 37 °C in the presence of test compounds. DNA decatenation was assessed by agarose gel electrophoresis.

### Budding yeast viability assay

For spot assays, the S. cerevisiae YFK30 strain (MATa TRP1 erg3::HIS3 pdr1::hisG pdr3::hisG pdr5::LEU2) and top2 K414N strain (MATa TRP1 erg3::HIS3 pdr1::hisG pdr3::hisG pdr5::LEU2 top2-K414N) were grown to a concentration of 2.5 × 10^7^ cells/ml in YPD medium (glucose 2%, yeast extract 1%, polypeptone 2%, adenine 0.04%, uracil 0.02%). Serial dilutions (1:10) were prepared, and aliquots (4 μl) were spotted onto plates containing the indicated concentration of drugs (MST-312 or doxorubicin); plates were then cultivated at 30 °C. All procedures were performed in accordance with the institutional guideline and regulation under approval by Hiroshima University Safety Committee for Recombinant DNA Experiments.

### siRNA-mediated knockdown

*hTERT* siRNA (5′-CATTCCTGCTCAAGCTGACTCGACA-3′) was designed by BLOCK-iT RNAi Designer (Thermo Fisher Scientific, Waltham, MA, USA). Negative control siRNA was Stealth RNAi Negative Control Medium GC Duplex #2. All siRNAs were purchased from Thermo Fisher Scientific and introduced into cells with the reverse transfection method using Lipofectamine RNAiMAX Transfection Reagent (Thermo Fisher Scientific), according to the manufacturer’s instructions.

### Plasmid transfection and Retroviral infection

pLNCX2/lamin C was generated by amplifying lamin C cDNA from total cDNA of A549 cells as a template and subsequently inserting it into the pLNCX2 vector (BD Biosciences). NCI-H522 cells were transiently transfected pLNCX2 or pLNCX2/lamin C with Lipofectamine 2000 transfection reagent (Thermo Fisher Scientific), according to the manufacturer’s instructions. pLNCX2/hTERT was generated by cloning the hTERT fragment^66^ into the pLNCX2 vector. pLHCX/lamin A was generated by cloning the lamin A fragment (Open Biosystems, Cambridge, UK) into the pLHCX vector (BD Biosciences). Amphotropic retroviruses were produced by transfecting the plasmids into Phoenix amphotropic cells using standard calcium phosphate precipitation. NCI-H522 cells were infected with the retrovirus essentially as described^67^. Infected cells were selected with 400 μg/ml of G418 and/or 200 μg/ml of hygromycin and cultured as described^68^. All procedures were performed in accordance with the institutional guideline and regulation under approval by the JFCR Safety Committee for Recombinant DNA Experiments.

### Statistical analysis

Unpaired *t* tests were performed for comparison of the groups with and without MST-312 treatment. Tukey-Kramer tests were performed to examine every combination of multiple experimental groups.

## Electronic supplementary material

Supplementary Figures

## Data Availability

The datasets generated and analysed during the current study are available from the corresponding author on reasonable request.
