# Supplementary material for: Cell-based chemical fingerprinting identifies telomeres and lamin A as modifiers of DNA damage response in cancer cells
Source: Sci Rep. 2018 Oct 4;8:14827. doi: 10.1038/s41598-018-33139-x (PMC6172206; doi:10.1038/s41598-018-33139-x)
Supplement: Supplementary file 1 — Supplementary Figures [file 41598_2018_33139_MOESM1_ESM.pdf]

Cell-based chemical fingerprinting identifies telomeres and lamin A as modifiers of DNA damage response in cancer cells

Chiaki Fujiwara, Yukiko Muramatsu, Megumi Nishii, Kazuhiro Tokunaka, Hidetoshi Tahara, Masaru Ueno, Takao Yamori, Yoshikazu Sugimoto, and Hiroyuki Seimiya

<u>Supplementary Figures S1 to S5</u>

Supplementary Figure S1.
Correlation between MST-312 sensitivity and telomerase activity.

Supplementary Figure S2.
Effect of lamin C expression on MST-312-induced DNA damage.

Supplementary Figure S3.
Effect of lamin C expression on telomerase activity.

Supplementary Figure S4.
Full scans of the immunoblots shown in Fig. 1G.

Supplementary Figure S5.
Full scans of the immunoblots and Coomassie-stained gels shown in Figs. 5 and 6.

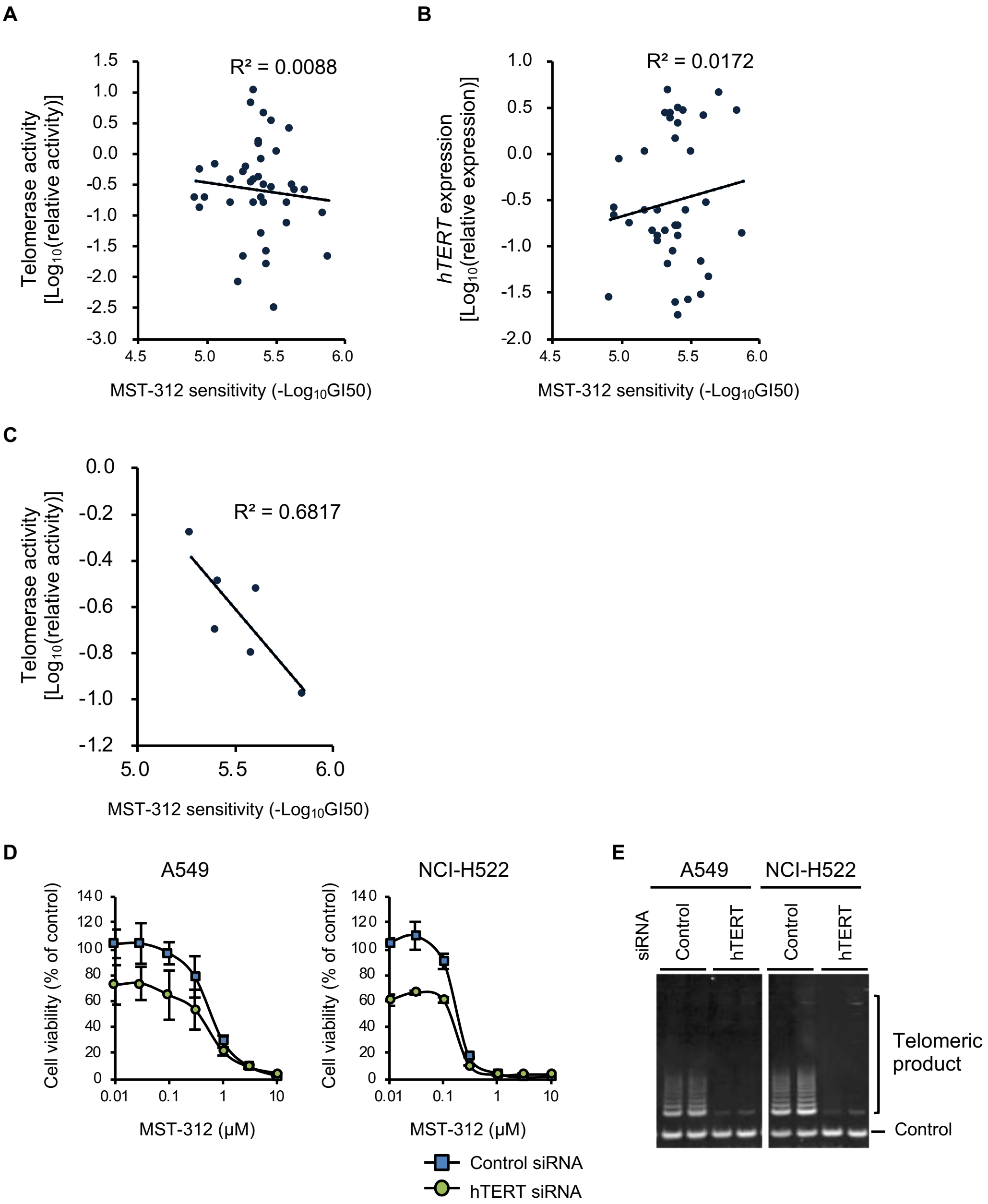

Supplementary Figure S1. Correlation between MST-312 sensitivity and telomerase activity. (A, B) Correlation between telomerase activity (A) or *hTERT* expression level (B) and MST-312 sensitivity in the JFCR39 cancer cell line panel. (C) Correlation between MST-312 sensitivity and telomerase activity in six cell lines, which have very short telomeres (PC-3, St-4, MKN45, MKN7, NCI-H522 and MKN74; mean TRF length < 4 kb). (D) Anti-proliferative effect of MST-312 on hTERT-depleted A549 and NCI-H522 cells. Cells were treated with indicated concentrations of MST-312 for 96 h and then cell proliferation levels were quantitated by MTT assay. Normalization was done with DMSO (vehicle)-treated control siRNA cells, which were defined as 100%. (E) Knockdown efficiency of hTERT in (D) determined by TRAP assay.

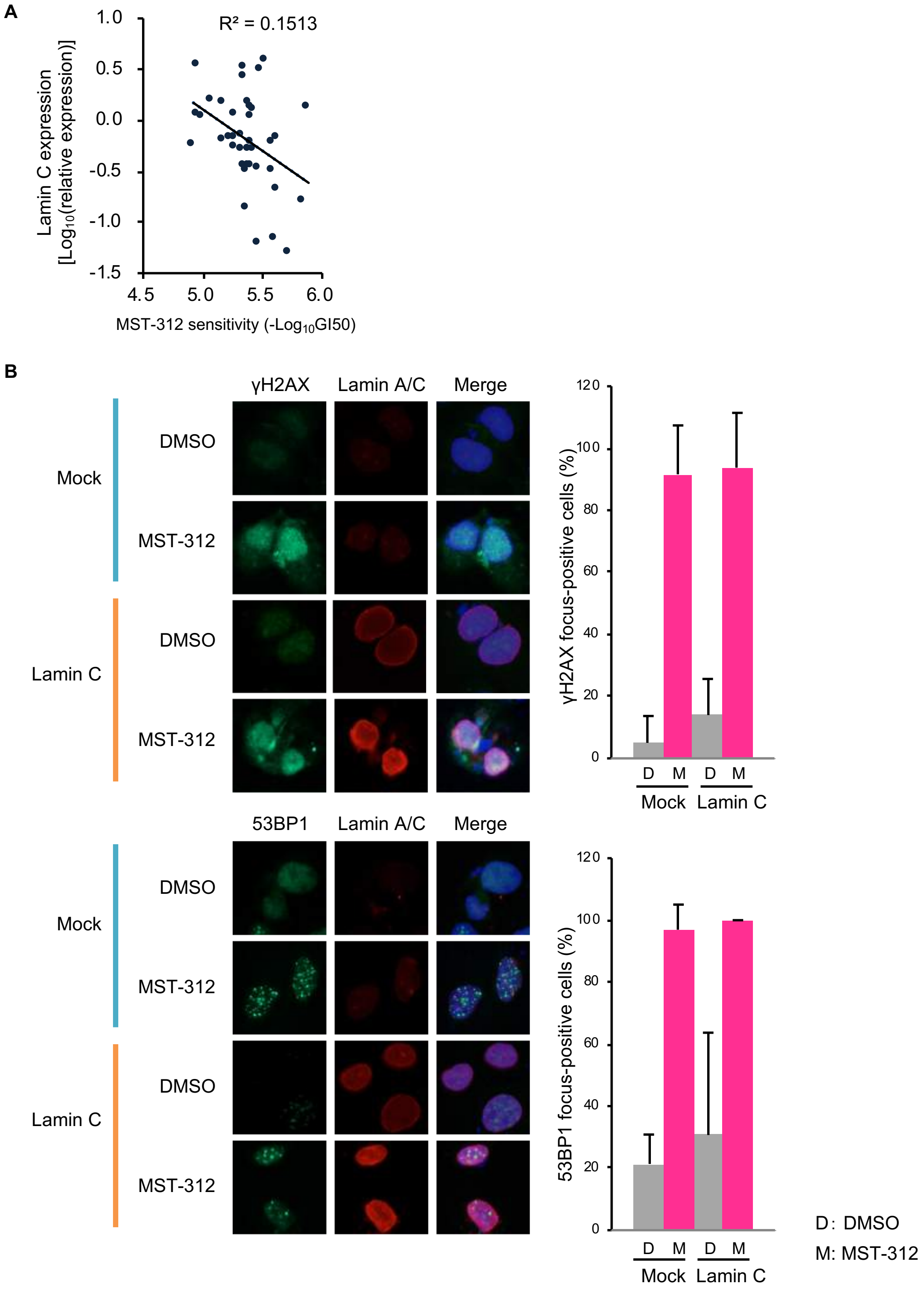

Supplementary Figure S2. Effect of lamin C expression on MST-312-induced DNA damage. (A) Correlation between lamin C protein expression level and MST-312 sensitivity in the JFCR39 cancer cell line panel. (B) Immunofluorescence staining with γH2AX (*upper*) and 53BP1 (*lower*) antibodies in NCI-H522 cells. Cells were transfected with pLNCX2 empty vector or pLNCX2-Lamin C vector and then treated with 5 µM MST-312 for 48 h. *Right*: Quantification of MST-312-induced DNA damage focus-positive cells. Cells with more than four γ-H2AX or 53BP1 foci were counted as positive.

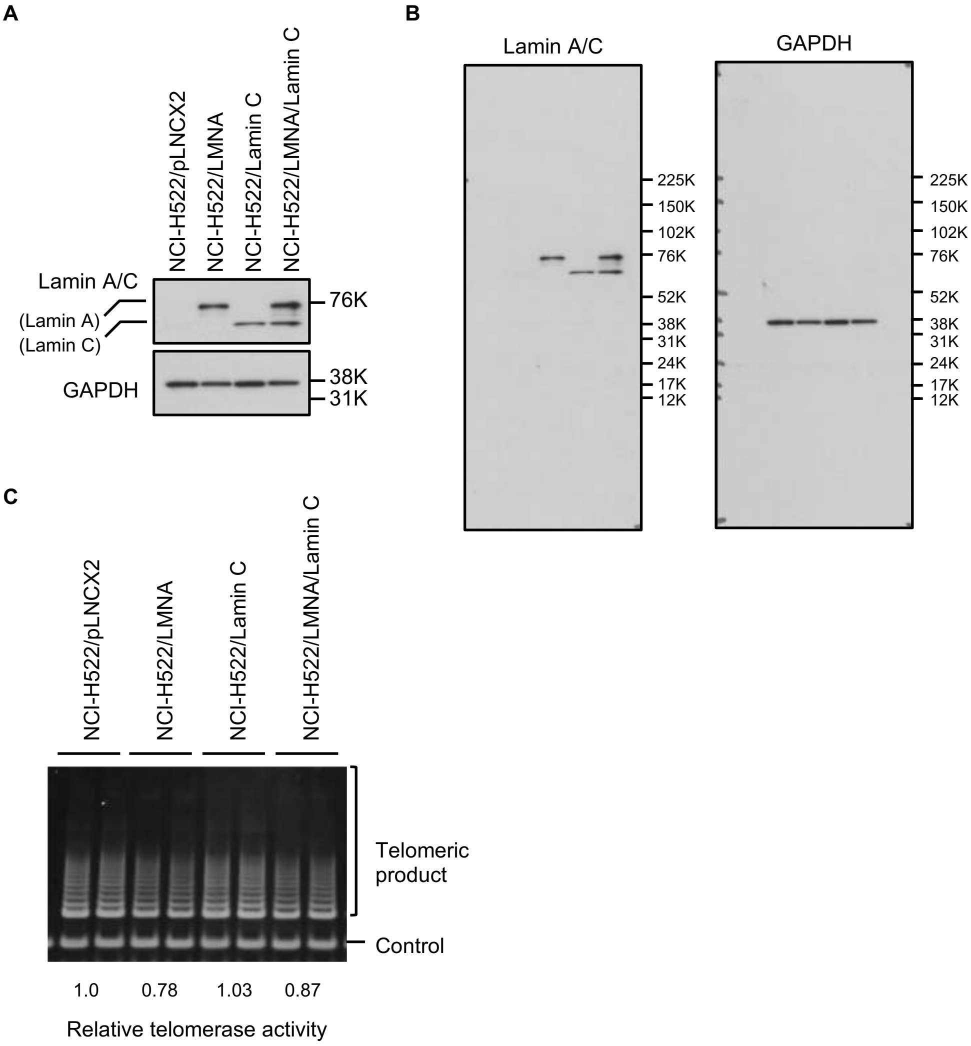

Supplementary Figure S3. Effect of lamin C expression on telomerase activity. (A) Western blot analysis to assess the ectopic expression of lamin C protein in NCI-H522/pLNCX2 and NCI-H522/LMNA cells. Cells were transfected with pLNCX2 empty vector or pLNCX2-Lamin C vector by lipofection. (B) Full-range blots of (A). (C) TRAP assay for detection of telomerase activity of the cells in (A).

Fig. 1G-1 (TRF1, TRF2, POT1)

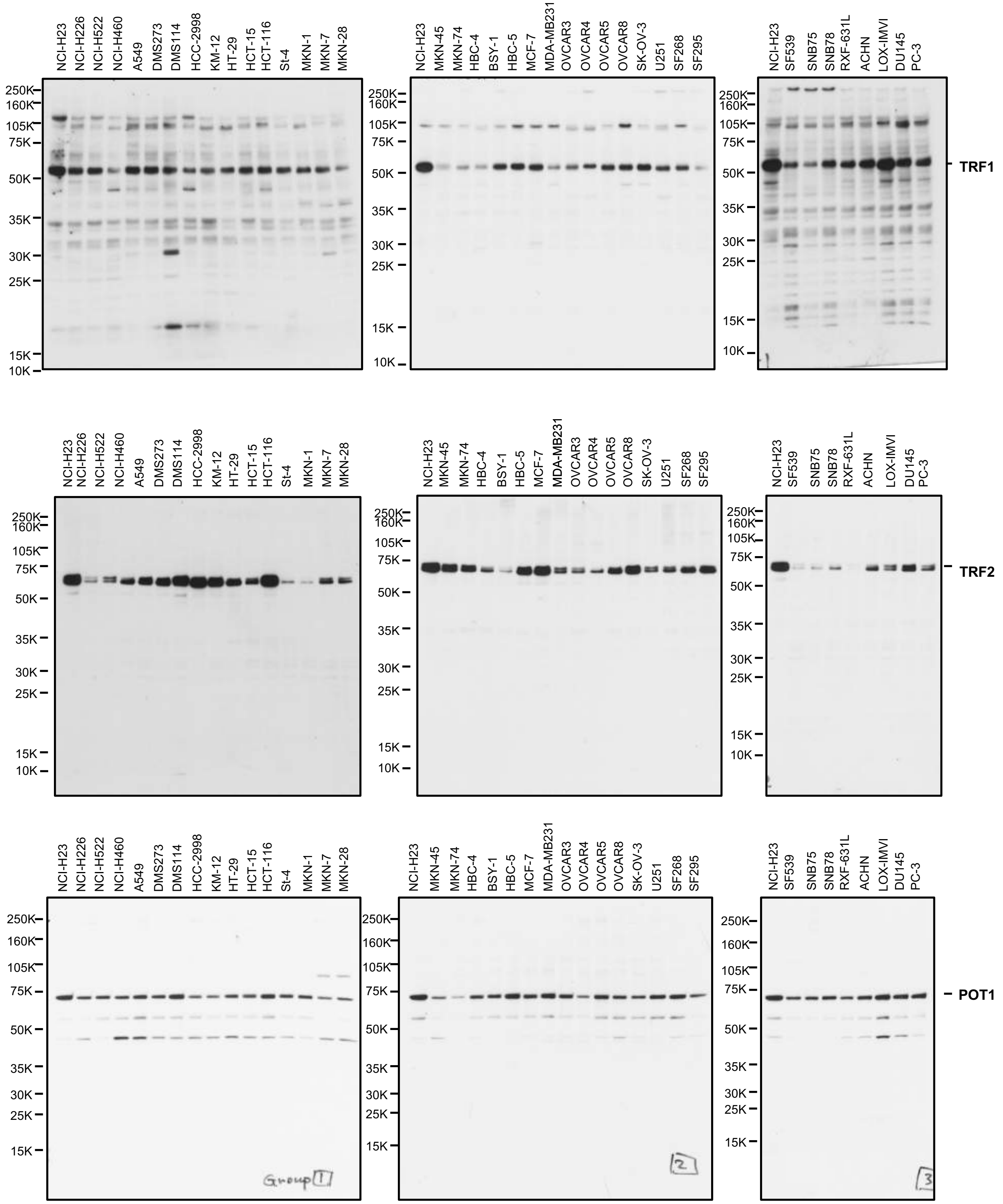

Fig. 1G-2 (TPP1, Rap1, TIN2)

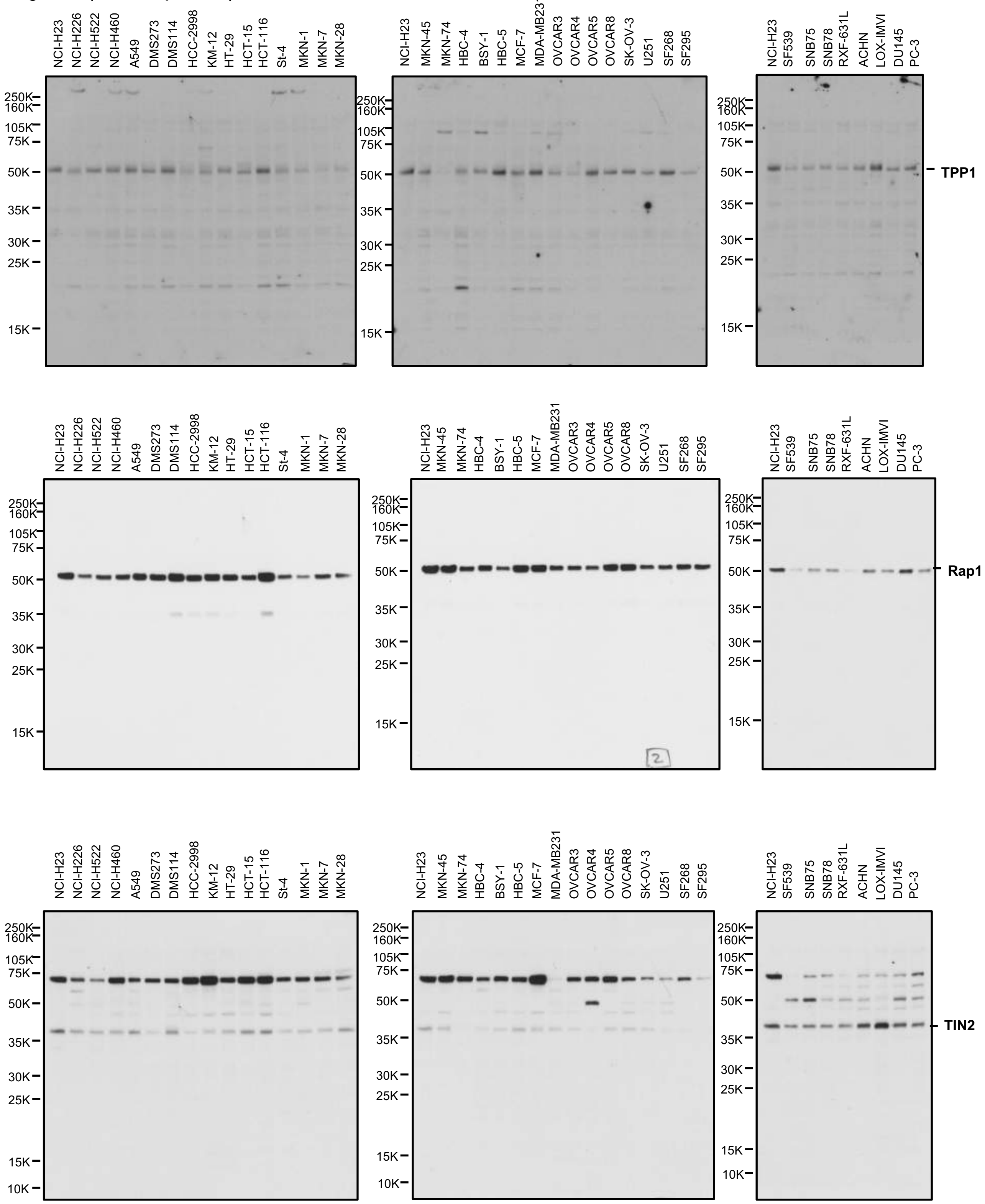

Fujiwara et al. Supplementary Figure S4 (*continued*)

Fig. 1G-3 (RIF1, Dyskerin, MRE11)

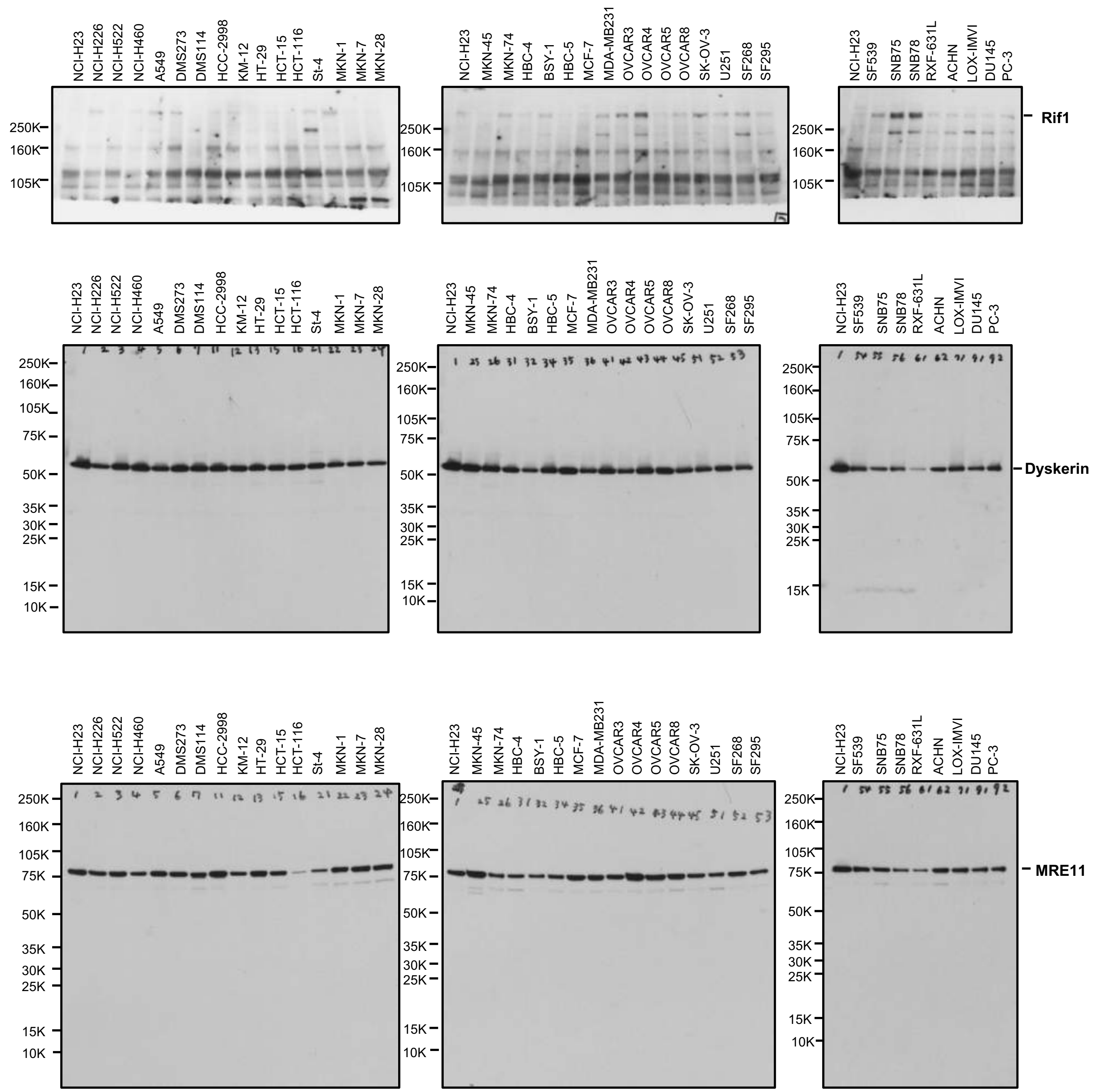

Fujiwara et al. Supplementary Figure S4 (*continued*)

Fig. 1G-4 (RAD50, NBS1, BLM)

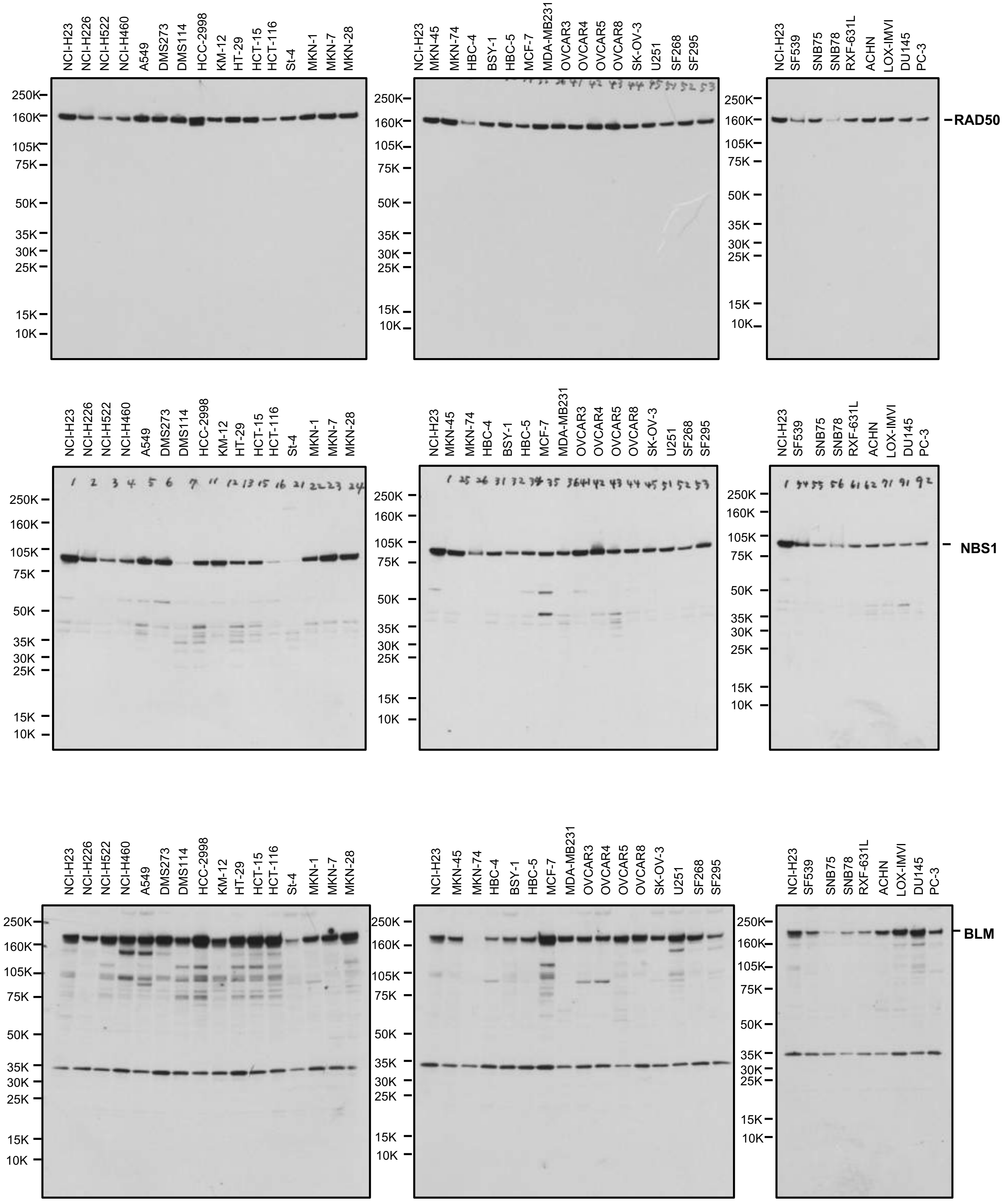

Fujiwara et al. Supplementary Figure S4 (*continued*)

Fig. 1G-5 (WRN, ATM, PARP-1)

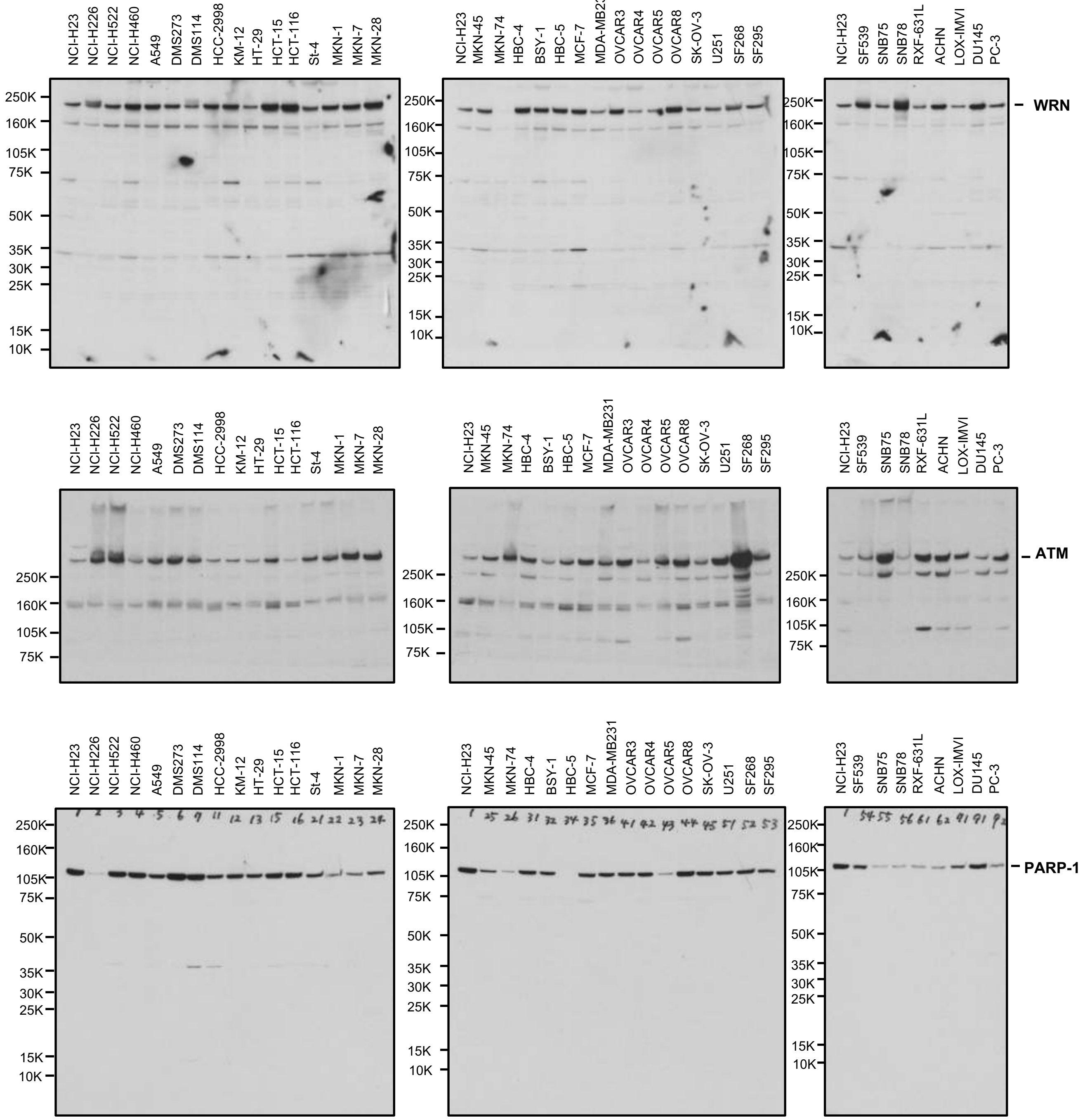

Fig. 1G-6 (Tankyrase-1, GAPDH, α-Tubulin)

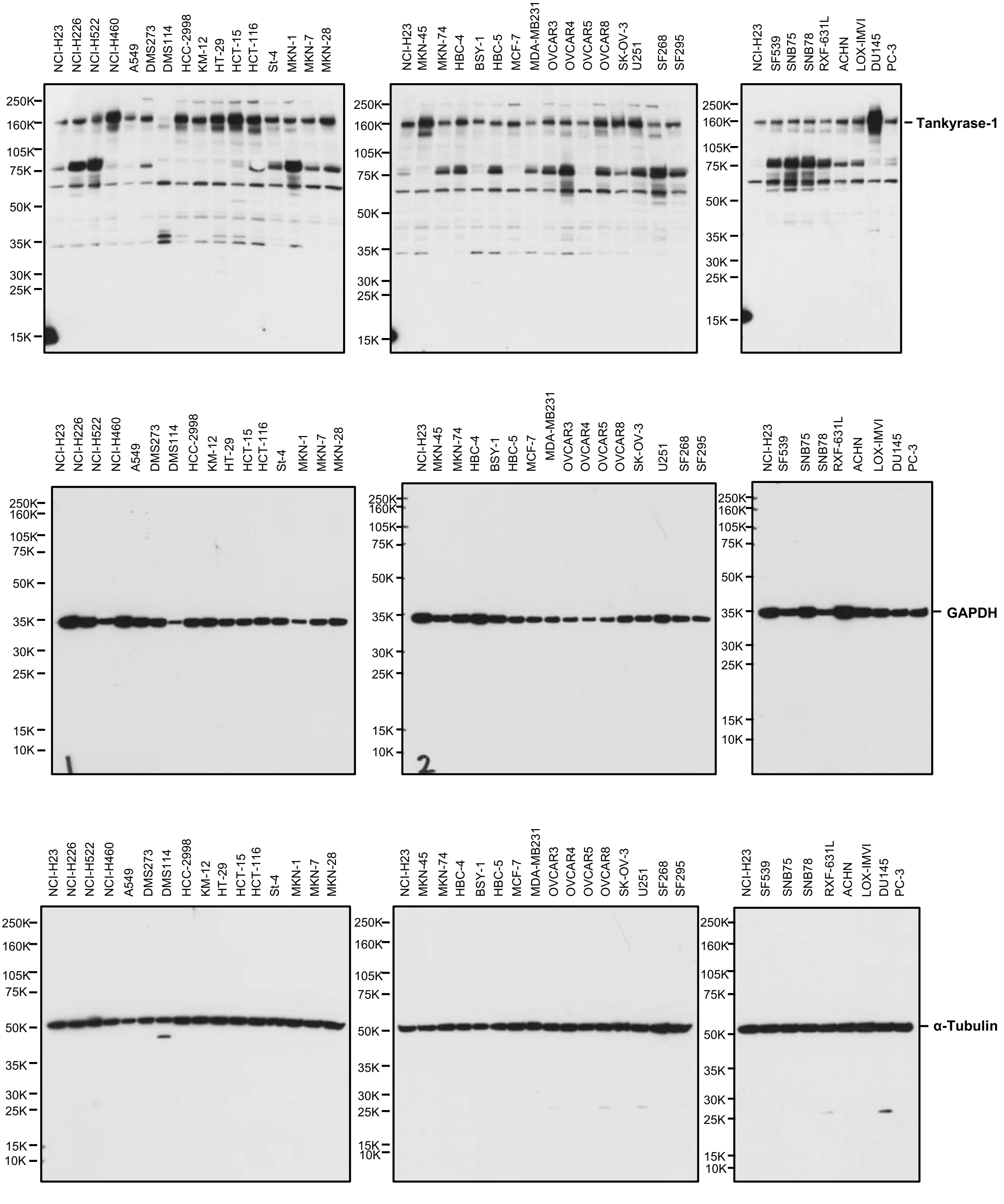

Fig. 5A **Lamin A/C**

Coomassie

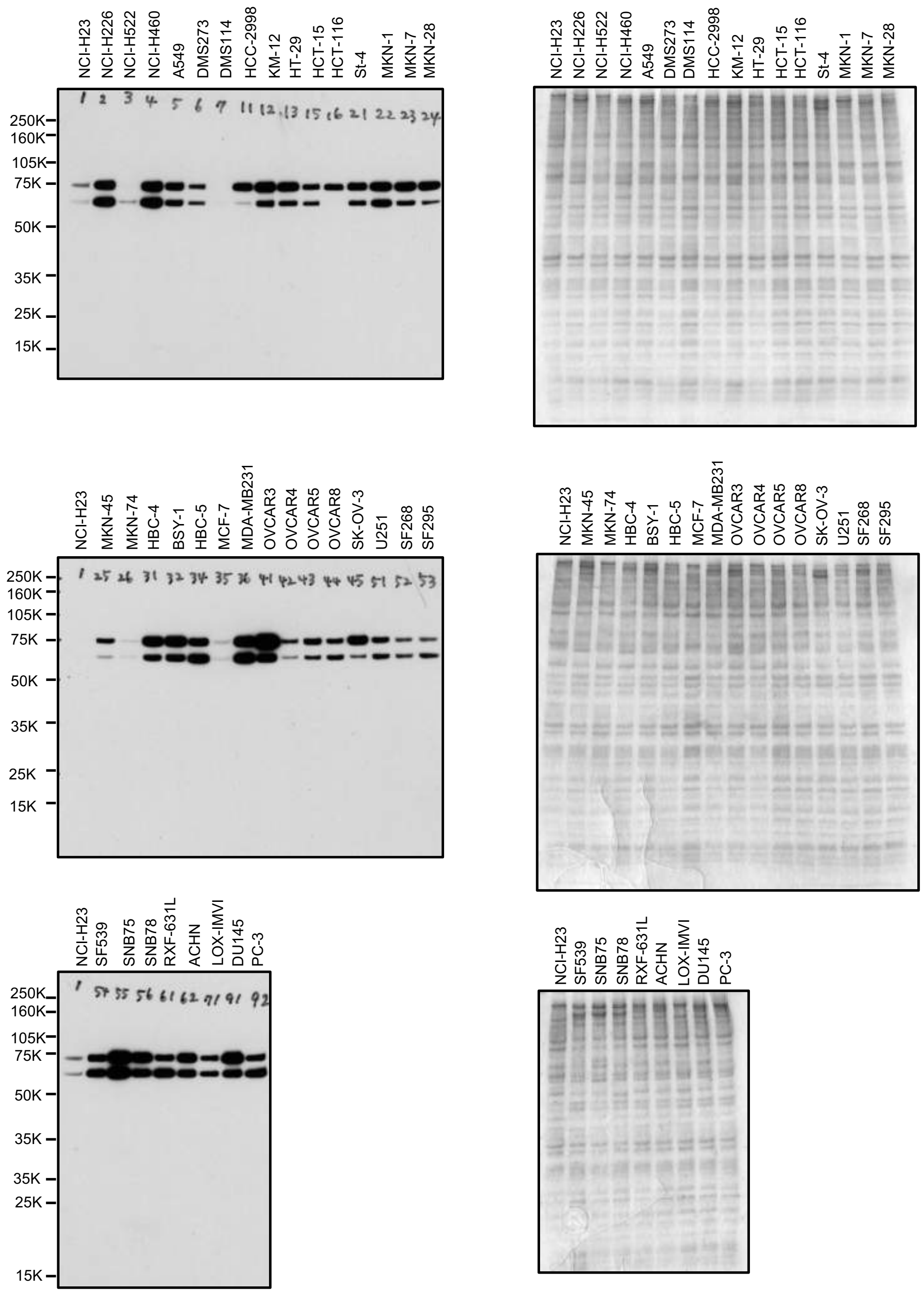

Fig. 5D

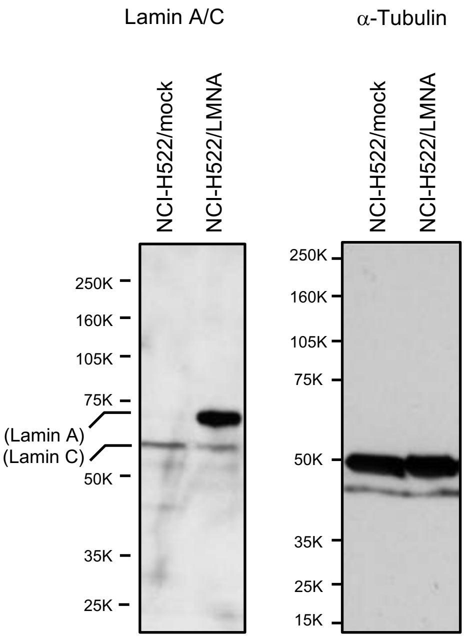

Fig. 6A

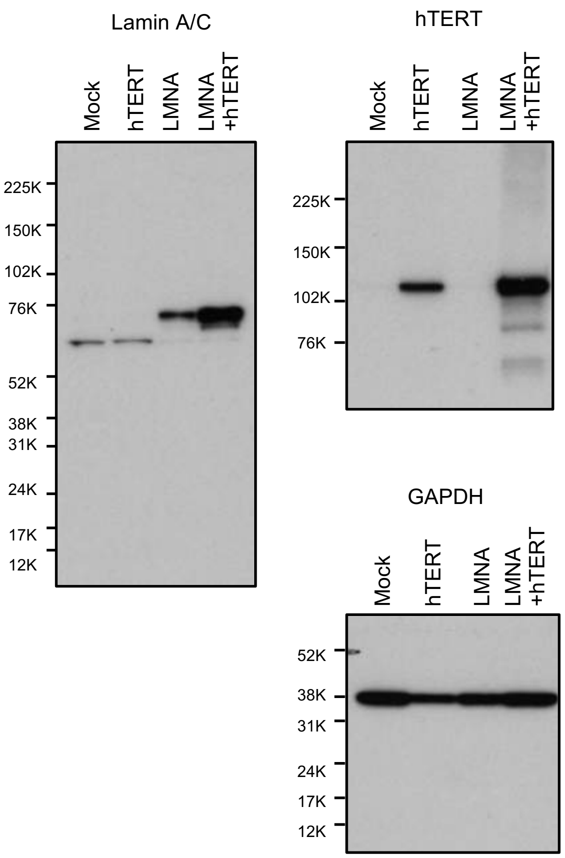

Fujiwara et al. Supplementary Figure S5 (*continued*)
